# An introductory biology research-rich laboratory course shows improvements in students’ research skills, confidence, and attitudes

**DOI:** 10.1371/journal.pone.0261278

**Published:** 2021-12-16

**Authors:** Iglika V. Pavlova, David L. Remington, Meg Horton, Elizabeth Tomlin, Mark D. Hens, David Chen, John Willse, Malcolm D. Schug

**Affiliations:** 1 Department of Biology, University of North Carolina Greensboro, Greensboro, North Carolina, United States of America; 2 Department of Educational Research Methodology, University of North Carolina Greensboro, Greensboro, North Carolina, United States of America; University of Mississippi, UNITED STATES

## Abstract

As part of a wider reform to scaffold quantitative and research skills throughout the biology major, we introduced course-based undergraduate research experiences (CURE) in sections of a large-enrollment introductory biology laboratory course in a mid-level, public, minority-serving institution. This initiative was undertaken as part of the in the National Science Foundation / Council for Undergraduate Research Transformations Project. Student teams performed two or three experiments, depending on semester. They designed, implemented, analyzed, revised and iterated, wrote scientific paper-style reports, and gave oral presentations. We tested the impact of CURE on student proficiency in experimental design and statistical reasoning, and student research confidence and attitudes over two semesters. We found that students in the CURE sections met the reformed learning objectives for experimental design and statistical reasoning. CURE students also showed higher levels of experimental design proficiency, research self-efficacy, and more expert-like scientific mindsets compared to students in a matched cohort with the traditional design. While students in both groups described labs as a positive experience in end-of-semester reflections, the CURE group showed a high level of engagement with the research process. Students in CURE sections identified components of the research process that were difficult, while also reporting enjoying and valuing research. This study demonstrates improved learning, confidence, and attitudes toward research in a challenging CURE laboratory course where students had significant autonomy combined with appropriate support at a diverse public university.

## Introduction

Student-centered, active learning approaches in higher education, including course-based undergraduate research experiences (CUREs) are widely accepted, high impact practices that enhance student success and retention in STEM disciplines [1, 2]. The 2011Vision and Change report from the American Association for the Advancement of Science called for a focus on student-centered learning as a campus wide commitment with engagement of the biology community nationwide [1].

CUREs represent one high-impact active learning practice that has been shown to improve undergraduate persistence and graduation with science, engineering, and math degrees and may be particularly beneficial to students from underrepresented groups (e.g., [2–5]). Research also demonstrates positive influences on student’s attitudes and perceptions about their learning, scientific skills, and the value of science [6–8]. The biology department at University of North Carolina Greensboro (UNCG) has been participating in the Council of Undergraduate Research (CUR) Transformations Project involving a total of 24 departments at 12 four-year higher education institutions representing a diversity of institutional types [9]. One of the major goals of the CUR Transformations Project is to support departmental reform by scaffolding CURE throughout the curriculum with a goal of guiding students toward greater independence and ownership of learning.

For this pilot study of Year 1 of reform, we implemented CURE modules focused on culturing independence in the design of experiments and the collection of data, the quantitative analysis and interpretation of results, and the communication of the findings in scientific-style reports and presentations. The design is consistent with the elements of CURE proposed by Auchincloss et al. 2014 [10]. It also supports two of our programmatic student learning outcomes (SLOs) for Biology degree programs, which were updated concurrently with our participation in the CUR Transformations Project:

- (SLO 1) Students will demonstrate proficiency in scientific inquiry, to include research design, hypothesis development, and analysis, interpretation and evaluation of biological data.
- (SLO 2) Students will demonstrate proficiency in research skills by carrying out investigations using appropriate techniques and equipment for biological research from the molecular to ecosystem levels.

CURE modules emphasized student autonomy, while also providing the support and structure necessary for students who are new to research. Research demonstrates that discovery, iteration, and collaboration dimensions in CURE are achieved through promoting student ownership [11]. Furthermore, promotion of student autonomy provides a venue for experiencing and persisting through challenges and failure that ultimately induce students to realize the experience of engagement in authentic research [12, 13].

The process of developing and implementing CUREs as part of curriculum reform is significantly influenced by faculty attitudes, resources, and structural issues in individual departmental curricula [14, 15]. In general, faculty need to be convinced that the effort to design CURE modules leads to positive impacts on student learning, persistence, and graduation in their own population of students, and that resources and support are available for change [16]. The number of laboratory sections in introductory and mid-level courses creates a logistic challenge to integrating undergraduate research and active learning into the curriculum in our department.

To prepare for the large-scale implementation of CURE in all sections of our introductory biology laboratories, we studied the impact of a CURE design on measures of student learning and attitudes in a subset of the BIO 112 laboratory sections for two semesters. We compared the learning and attitudes with those of students in which the traditional question-answer style design was still used. There has been a call to include metrics that assess student ability in CURE directly, rather than to rely primarily on student self-assessment of one’s own ability [7, 17]. We therefore included several measures of student achievement using published instruments as well as evaluation of laboratory reports and exams in the CURE sections. This approach provides a mechanism to analyze metrics relevant to both departmental reform and experimental design skills and statistical reasoning more generally [18–21]. We also included metrics for student research interest, confidence, and scientific mindsets because students’ subjective experiences have been shown to be important for learning and academic success and STEM career choice [11, 22–25].

We hypothesized that students who engage in research early in their courses have improved science-related skills and more positive attitudes toward research compared to students who do not. We predicted that students in the CURE laboratory sections would: 1) Meet the CURE-specific course learning objectives for experimental design and statistical reasoning; 2) Demonstrate higher scores on assessments and surveys for research interest, confidence, and scientific mindsets than students enrolled in traditional laboratory sections; and 3) demonstrate greater proficiency in meeting Biology program SLOs for scientific inquiry and research skills than students enrolled in traditional laboratory sections, as measured by assessments of experimental design and statistical reasoning skills.

## Methods

This study was conducted with approval from the UNCG Institutional Review Board under #17–0344 with exempt status and written consent.

### Study context and design

The demographic characteristics of students at the institution and in the biology major are presented in Table 1. UNCG is a public university with a Carnegie classification of R2: High Research Activity and Minority Serving Institution with approximately 20,000 students and 1300 biology majors. Biology offers two introductory majors level courses, BIO 111 Principles of Biology 1 (Cell and Molecular), and BIO 112 Principles of Biology 2 (Evolution and Ecology). BIO 111 and BIO 112 are required courses for all biology majors. They also serve majors from other science programs and non-science majors who are fulfilling general education requirements. BIO 111 typically enrolls approximately 1,000 students in the fall semester (including many non-biology majors in other health-related programs) with a corresponding 40 lab sections. BIO 112 enrolls approximately 330 in the fall semester with 16 corresponding lab sections

**Table 1:** Demographic information for the students in CURE sections, the propensity-score matched students in traditional sections, UNCG undergraduate biology majors overall, and UNCG undergraduates overall from the 2017–18 academic year.

	Fall 2017		Spring 2018					
	Traditional	CURE	Traditional	CURE	UNCG Biology		UNCG Overall	
Students	43	43	40	40	1,121		16,439	
Sex								
Male	19 (44%)	16 (37%)	10 (25%)	11 (28%)	295 (26%)		5,592 (34%)	
Female	24 (56%)	27 (63%)	30 (75%)	29 (72%)	826 (74%)		10,847 (66%)	
Race/Ethnicity								
Black	14 (33%)	15 (35%)	15 (38%)	12 (30%)	369 (33%)		4,652 (28%)	
White	21 (49%)	18 (42%)	13 (33%)	17 (43%)	408 (36%)		7,920 (28%)	
Other:	8 (18%)	10 (23%)	12 (30%)	11 (28%)	344 (31%)		3,867 (24%)	
*Hispanic*	*1 (2%)*	*2 (5%)*	*7 (18%)*	*5 (13%)*	*130 (12%)*		*1*,*503 (9%)*	
*Asian*	*3 (7%)*	*6 (14%)*	*2 (5%)*	*2 (5%)*	*127 (11%)*		*883 (9%)*	
*AA/AN* ^*1*^	*1 (2%)*	*--*	*1 (3%)*	*--*	*7 (1%)*		*64 (<1%)*	
*NH/PI* ^*2*^	*--*	*--*	*--*	*--*	*--*		*10 (<1%)*	
*2 or more*	*2 (5%)*	*2 (5%)*	*2 (5%)*	*4 (10%)*	*63 (6%)*		*787 (5%)*	
*Non-res*. ^*3*^	*--*	*--*	*--*	*--*	*10 (1%)*		*366 (2%)*	
*Unknown*	*1 (2%)*	*--*	*--*	*--*	*7 (1%)*		*254 (2%)*	
Class								
Freshman	20 (47%)	22 (51%)	20 (50%)	16 (40%)	328 (29%)		3,685 (22%)	
Sophomore	11 (26%)	7 (16%)	11 (28%)	10 (25%)	206 (18%)		3,279 (20%)	
Junior	10 (23%)	11 (26%)	6 (15%)	12 (30%)	304 (27%)		4,369 (27%)	
Senior	2 (5%)	3 (7%)	3 (8%)	2 (5%)	283 (25%)		4,656 (28%)	
Unclassified	0 (0%)	0 (0%)	0 (0%)	0 (0%)	0 (0%)		45 (3%)	
Course Grade ^4^					NA		NA	
A	10 (23%)	9 (21%)	12 (30%)	10 (25%)				
B	10 (23%)	12 (28%)	12 (30%)	14 (35%)				
C	13 (30%)	12 (28%)	11 (28%)	11 (28%)				
D/F	10 (23%)	10 (23%)	5 (12%)	5 (12%)				
First Generation								
Yes	15 (35%)	17 (40%)	24 (60%)	23 (58%)	463 (41%)		6,229 (38%)	
No	20 (47%)	14 (33%)	16 (40%)	17 (42%)	658 (59%)		10,210 (62%)	
Missing	8 (19%)	12 (28%)	--	--	--		--	
Pell Eligible								
Yes	26 (61%)	28 (65%)	23 (58%)	22 (55%)	642 (57%)		8,352 (51%)	
No	17 (40%)	15 (35%)	17 (42%)	18 (45%)	449 (43%)		8,087 (49%)	
Mean age (sd)	19.86	20.23	19.70	20.10	NA		NA	
	(2.31)	(3.37)	(3.86)	(3.64)				
Mean credit hours enrolled (sd)	14.60	14.81	14.85	14.33	11.49		13.12	
	(1.61)	(1.81)	(2.21)	(3.42)	(NA)		(NA)	
Grade point average (sd)	2.89	3.23	2.95	3.04	NA		NA	
	(0.81)	(0.61)	(0.90)	(0.94)				

^1^ American Indian or Alaska Native.

^2^ Native Hawai’ian or Pacific Islander.

^3^ Non-resident alien.

Students were recruited for the study in the Fall 2017 and Spring 2018 semesters in a quasi-random design as the students had no prior knowledge of which sections would have CURE modules (three CURE-based sections in both semesters) or traditional instruction (11 sections in Fall 2017, 12 sections in Spring 2018). This design addressed possible selection bias for one design compared to the other. To avoid study participation bias, all students regardless of consent choice completed the surveys which were incorporated as part of coursework in both curricular modes (CURE-based and traditional). To minimize instructor bias, a research assistant who was not an instructor in the course managed survey collection and instructors were blinded to student consent status. Surveys were blinded to the instructors during the study. Students were made aware of confidentiality in the recruitment email and study consent forms.

From the 539 students that completed the Pre-Survey and consent process in Fall 2017 and Spring 2018 semesters, 461 students consented (91%) and completed both the Pre and Post surveys (Fall 2017, *n* = 208; Spring 2018, *n* = 218) and were included in the final study. Propensity score matching was used to create a matched comparison group for participants in CURE instruction sections (Fall 2017, *n* = 43; Spring 2018, *n* = 40) from those in the traditional instruction sections (described below in Statistical analysis). Analyses from Pre and Post surveys are reported for matched traditional and CURE participants; additional analyses comparing CURE to all traditional design participants are provided in the Supporting Materials. For open-ended measures that were administered in both groups (E-EDAT and reflections), only data from matched groups were coded and reported (described below in Coding of written work).

### Traditional and CURE designs

The traditional and CURE course designs were implemented over 13 weeks in the semester. Lab sessions lasted 3 hours in the same laboratory room capped at 24 students and one instructor per section. CURE sections were taught by teaching faculty and traditional sections were taught by either graduate students or teaching faculty. Students worked at tables of four, with a pair of students on each side of the shared table.

The traditional design consisted of prepared laboratory activities with a different topic each week in the areas of ecology, evolution, and organismal diversity (S1 File). Students were guided during each session to engage in laboratory skills (such as pipetting and calculations) using a highly structured lab manual that had extensive questions to answer. The traditional design included limited structured experiences in hypothesis development, data analysis, and data interpretation, and did not include background research, designing experiments, statistical analyses nor data presentation (Table 2). For example, students were asked to develop hypotheses before they collected data, they collected and graphed data, and answered questions on the experimental results or based on other observations (such as from microscopic examination or dissection). Students who sat at the same table could work together (for example, during dissections) and discuss answers. However, collaboration was not required or necessitated by most tasks. Overall, the traditional design did not include the five elements of CURE described by Auchincloss and colleagues [10] and can be described as level ½ of inquiry in the model proposed by Buck and colleagues [26].

**Table 2:** Comparison of research elements in the traditional and CURE course designs.

	Research elements	Traditional	CURE
1	Background research and observations	Not included.	Students perform background research using primary and secondary sources and perform preliminary observations or experimentations for each experiment.
2	Develop hypothesis	Students develop hypotheses based on information in the lab manual in six out of nine lab sessions.	Students develop hypotheses based on background research and observations for each experiment. Students defend the rationale for their experiment, linking to the broader significance of experiment.
3	Design an experiment	Limited. Students choose what to test and where to collect in one lab. No iteration.	Students design each experiment based on background research and observations, to include choice of variables, choice of control or comparison groups, and design of the experimental protocol. Design is iterative, with students revising the design for each experiment based on team and class discussions, and experimental results.
4	Perform experiment	Students perform tests for the hypotheses. No iteration.	Students perform experiment and iterate experiment after evaluation.
5	Data collection and recording	Students collect data or perform computer simulations. Some data are plotted graphically in the lab manual. No iteration.	Students collect data and record in Excel tables and graphs. Students make decisions on how to record and graph data. Students improve tables and graphs in preparation for the lab report.
6	Data analysis and interpretation	Students perform algebraic calculations and formulate conclusions based on collected data.	Students are instructed in statistics and perform descriptive and inferential statistical analyses. Students formulate conclusions based on consideration of statistical analysis, confounding variables, and experimental limitations.
7	Presentation	Not included.	Students prepare a lab report for each experiment over several weeks. Student teams present their research to other teams for one experiment and present to entire class for another experiment.

The CURE course learning objectives are research-focused, specifically stating that students will:

1. Perform background research and develop hypotheses.
2. Design experiments.
3. Learn and practice laboratory skills.
4. Record and analyze quantitative data, including gaining proficiency in using Excel.
5. Perform statistical significance testing.
6. Evaluate experimental results to suggest improvements to the experimental design or to answer further questions stemming from the results.
7. Perform improved experiments or further exploration based on evaluation of first experiment.

In the CURE-based sections, the modules implemented research elements in every lab period including background research, hypothesis development, designing experiments (to include choice of variables, choice of control or comparison groups, and designing experimental protocol), data analysis, data interpretation, and data presentation in written and oral form (Table 2). The CURE design was piloted in Spring 2017 [27] and included all elements of research methods, statistical analysis of results and interpretation of quantitative data and statistical analysis. The design presented here describes the subsequent Year 1 of curricular reform under the CUR Transformations Project. Students completed three (Fall 2017) or two (Spring 2018) experiments under a unifying theme of plant-herbivore interactions (syllabi are provided in the S2 File). The experiments included investigations of 1) factors affecting diversity of leaf-litter invertebrates in a campus park (both semesters), 2) the effect of bioactive compounds on caterpillars (both semesters) and 3) on *Daphnia* (Fall 2017 only).

The CURE format was designed to engage students in research that moved beyond the inquiry instructional model and into a CURE model [10]. To emphasize the elements of discovery, multiple scientific practices, and collaboration, each of the experiments provided students with a general area of study within which they developed their research. Students worked in groups performing background research for all experiments to determine the rationale for an experiment of their choice to study unanswered questions with the findings unknown to them and the instructor, and with potential value to the larger scientific community. Students explored the less-studied terrestrial leaf-litter ecosystem in the diversity experiment. They also investigated the effect of bioactive compounds of their own choice on ecologically important species (caterpillar and Daphnia) in the other two experiments. *Daphnia* is commonly used as a bioindicator species [28]. Students were aware of the larger significance of their experiments through their background research of published studies. To answer their research questions, students collaborated with their peers and instructors at all stages of the research process, starting with background research and experimental design to perform the experiments, analyze their data, and iterate on their experiment based on experimental findings to collect more data or improve the experimental design. Special emphasis was placed on students experiencing the strengths and limitations of different experimental choices, with students allowed to make their own decisions and to think critically about the impact of their choices.

The CURE design also emphasized student autonomy in experimental design and iterative experimentation [10, 29]. In this respect, the invertebrate biodiversity experiment was the most structured module. Students were given the dependent variable to measure (invertebrate biodiversity in leaf litter) and they chose the independent variables and identified the comparison groups (e.g., leaf litter under native versus non-native trees). Students visited the UNCG main campus Peabody Park before they designed their experiment to explore the ecosystem and think of possible research questions, and to measure abiotic variables.

In the caterpillar bioactive compound experiment, students chose the independent and dependent variables and designed the experiment, with two iterations. Specifically, students decided which compounds to present to the caterpillars, the mode of presentation and the measurement variables (e.g. how much food was eaten, caterpillar growth, survival, feeding behavior and time to pupation). After evaluating the results students modified their approach and conducted a second experiment. For example, they might have modified how the food choices were presented, how many caterpillars were used, or varied the test compound dosages.

In the *Daphnia* bioactive compound experiment (Fall 2017 only), students iterated the experiment three times, establishing the protocol for the first two experiments to evaluate potential biases and confounding factors. In the first iteration, students typically discovered that *Daphnia* heart rate measurements are imprecise, and that experimenter bias is a significant issue. In the second iteration, students modified their protocol based on their first experiment to reduce bias and to measure heart rate more accurately. In the third iteration, they used their protocol to test the effect of compounds of their choice on heart rate in *Daphnia*.

### Measures

Students completed Pre-Post Surveys as part of regular coursework; a Pre-Survey at the start of the semester and a Post-Survey at the end of the semester (S3 File). Only the Pre-Survey included demographic questions. Both the Pre and Post Surveys included the SRBCI, E-EDAT, and CLASS-Bio instruments, and questions on research skills and STEM interest. The Post-Survey also included the end-of-semester reflections. A lab report and the lab exam were collected from the CURE group for analysis.

#### Lab report

Students in the CURE sections completed a lab report for each of the experiments. The *Daphnia* and Caterpillar lab reports (Fall 2017 and Spring 2018, respectively) were collected for analysis (S4 File).

#### Lab exam

Students in the CURE sections completed a lab exam which consisted of 20 questions, a combination of true-false, multiple-choice, and short answer questions on experimental design and statistical reasoning (S5 File).

#### Colorado Learning Attitudes about Science Survey for Biology (CLASS-Bio)

The CLASS-Bio [30] is a 31-item forced-choice pre-post-test instrument used to measure novice-to-expert-like perceptions about biology. Students are asked to rate their agreement to each survey statement such as “I think of biology in my everyday life”. Each item is assessed on a five-point Likert-type scale ranging from ‘strongly agree’ to ‘strongly disagree’, and responses are scored based on whether they agree with the response of biology experts. CLASS-Bio prompts are grouped in seven different areas, namely real-world connections, problem-solving difficulty, enjoyment, problem-solving effort, conceptual connections, problem-solving strategies, and reasoning.

#### Research skills questionnaire

We developed a 14-item questionnaire on student confidence related to common research skills, ranging from performing background research, experimental design and analysis, to collaborative work (S3 File). The questionnaire uses a five-point Likert-type scale ranging from “not confident” to “extremely confident.”

#### End-of-semester reflections

Reflections administered at the end of the semester ask students to respond in writing to five prompts on what they found most interesting (prompt 1), most valuable (prompt 2) and most difficult (prompt 3) in the lab in the past semester, as well as what changed the most about how they think and feel about the process of science (prompt 4) and about undergraduate science labs (prompt 5) (S3 File).

#### Statistical Reasoning in Biology Concept Inventory (SRBCI)

The SRBCI [21] is a 12-item forced-choice pre-post-test instrument designed to measure students’ statistical reasoning ability in biology. Items include assessing conceptual understanding of statistical variation, analysis of graphs and tables, and interpretation of results.

#### Expanded Experimental Design Ability Tool (E-EDAT)

The E-EDAT [18, 20] is an open-ended pre-post-test response instrument in which students design a strategy to address a company’s claim regarding a particular product. Students are asked to design an experiment and to justify their experimental choices. A scoring rubric is used to assign points to each of the elements of the E-EDAT.

### Coding of written work

Written responses for the lab report, lab exam, E-EDAT, and reflections were coded by two researchers (a pair for each response amongst IP, ET, and MH) blinded to student and group identity (traditional versus CURE, Pre versus Post) according to coding schemes (S6–S8 Files). The lab report was scored for experimental design and statistics criteria according to a rubric developed based on the Rubric for Experimental Design (S6 File, [19]). The written questions on the lab exam were scored according to a rubric we developed based on the learning outcomes for major concepts in experimental design and statistics for the course (S7 File). The E-EDAT was scored according to a slightly modified version of the published rubric (S8 File). The reflections were coded for emergent themes in student responses. Interrater reliability for the E-EDAT, reflections, lab report, and lab exam was assessed by examining Pearson correlation coefficients (*r*) for each of three pairs of coders (E-EDAT) or two coders (lab report, lab exam, and reflections). Correlation coefficients were found to be acceptable (range, 0.72–0.89); the scores were then averaged for further analysis (S10 File).

### Propensity score matching

Linear regression was run to investigate any pre-existing student differences between the traditional and CURE sections. The following covariates were examined: sex, age, race/ethnicity, Pell eligibility, credit hours enrolled for the semester, class membership by number of credits earned (i.e., Freshman, Sophomore, Junior, Senior), and course grade. Although groups did not statistically differ according to any of the covariates above, propensity score matching was used to create a matched sample of students in traditional vs. CURE sections, for each of the two semesters.

Propensity score matching (PSM) is used to control for the effects of potential confounding variables to provide an unbiased estimate of the effect of the treatment (i.e., CURE vs. traditional design) [31]. Thus, outcomes can be more accurately observed when the impact of differing group characteristics has been reduced. An attractive feature of PSM is that it uses the entire sample to find students from each group that most closely resemble each other in terms of characteristics. The R ‘MatchIt’ package [32] was used to perform PSM. Optimal matching was chosen to find matched pairs of students with the smallest average absolute distance across all matched pairs.

To check the PSM results, propensity scores and the absolute standardized difference in means for each of the covariates were analyzed before and after matching. A total of ten students in the CURE sections in Fall (one student) and Spring (nine students) were not used in the match because some demographic variables were not available. Demographic information for the Fall and Spring matched samples are provided in Table 1.

### Statistical analyses

The data analyzed included student scores on outcome measures taken at the beginning and end of the semester, college GPA, and demographic information such as sex, race/ethnicity, and age. Demographic information was collected from the university’s Office of Institutional Research. All analyses were run using SPSS 20.0 [33].

Average Pre-Post SRBCI score and CLASS-Bio percentage differences for traditional and CURE participants were analyzed with an independent samples *t*-test and the Wilcoxon signed-rank test when data failed to meet assumptions of normality or homogeneity of variances. Effect sizes were evaluated using Cohen’s *d*, which estimates the difference in means between the two groups as a fraction of the pooled standard deviation within the groups [34]. For the CLASS-bio, a one-way MANOVA was also run to examine differences between the two groups on the set of seven factors. Effect sizes were analyzed using partial η^2^, which measures the proportion of variance explained by the group effect after correcting for effects of other variables in the model.

Data from the research skills questionnaire were analyzed with a two-way MANOVA to evaluate the effects of group, semester, and their interaction on the multivariate response to the 14 questions. Separate one-way MANOVAs were also used to analyze each semester separately. With both the combined and individual-semester data, group effects were also analyzed for each individual question.

Data from the E-EDAT instrument were analyzed with a repeated measures ANOVA was run to examine the main effect of time (Fall or Spring) and group (traditional or CURE), as well as interaction between time and group on average pre-posttest differences. For the reflections, an independent samples *t*-test was run to examine differences between traditional and CURE average scores. Pearson’s correlations (*r*) between the average lab report score and Pre/Post E-EDAT scores were analyzed, as well as between the lab exam score and the SRBCI, E-EDAT, and lab report scores.

## Results

### Did CURE students meet the course learning objectives for experimental design and statistical analysis?

#### Lab reports and lab exam

**Fig 1:**
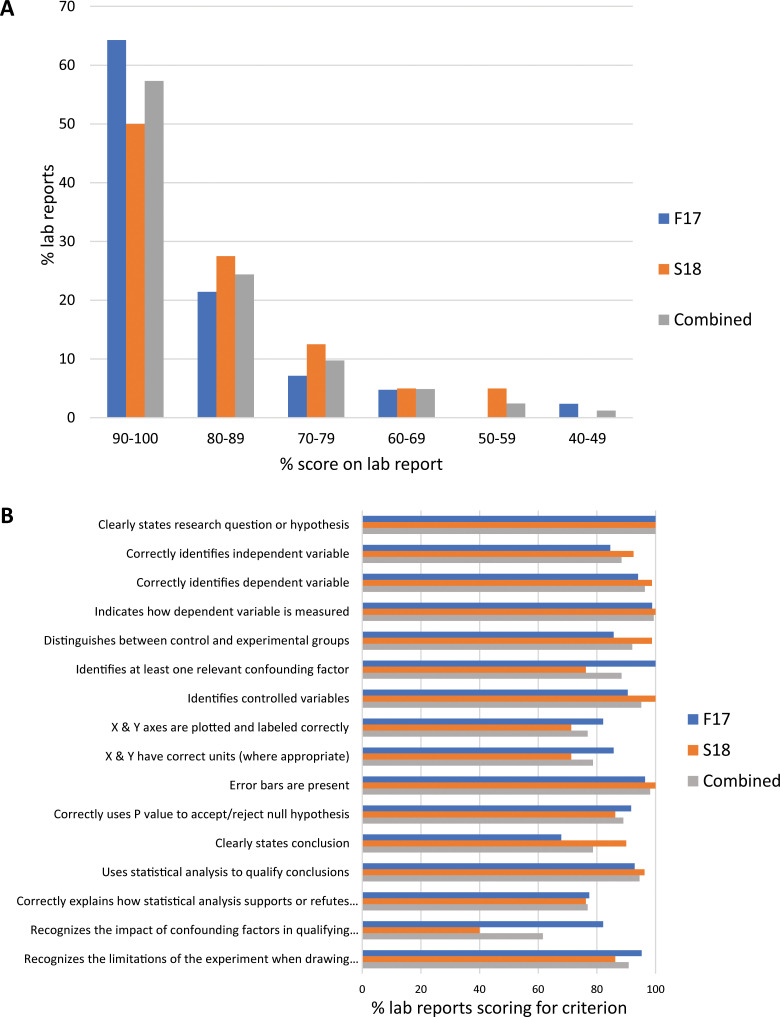
Lab report scores in the CURE sections. Most students in CURE sections scored high on criteria for experimental design, graphing, statistical analysis, and evidence-based conclusions on the lab report. A. Percentage of lab reports by the combined total score on all 16 criteria. B. Percentage of lab reports scoring as meeting each of 16 criteria. Fall 2017, *n* = 42 CURE. Spring 2018, *n* = 40 CURE. “Combined” group represents combined data from Fall 2017 and Spring 2018, *n* = 82 CURE.

The lab report was scored on whether the students met 16 binary criteria on experimental design, graphing, statistical analysis, and evidence-based conclusions (S6 File). Based on typical grading practices, we treated scores of 70% of criteria met as a lower bound for proficiency in learning outcomes. From the total scores on the lab reports among the two semesters, 91.5% of students scored 72% or higher, 82% achieved scores of 81% or higher, and 57% achieved scores of 91% or higher (Fig 1A). Students in the Fall 2017 semester scored slightly higher (93% students with ≥ 72%, 86% with ≥81%, and 64% with ≥ 91%) than students in the Spring 2018 semester (90% students with ≥ 72%, 78% with ≥81%, and 50% with ≥ 91%). We also calculated the percentage of students meeting each of the different criteria in order to evaluate areas of relative strength and weakness. Students performed most strongly in areas of clearly and correctly identifying the research question (criteria 1–4) and explaining controlled experimental design (criteria 5–7) (Fig 1B). Performance was also strong in some areas of statistical reasoning and evidence-based conclusions (criteria 11–16), specifically using statistical analysis as part of the conclusions and demonstrating how the limitations of the experiment impact the conclusions (Fig 1B). Some students struggled with graphing, statistical reasoning, and evidence-based conclusions, specifically being able to interpret how the statistics and confounding variables impact the conclusions, based on relatively low percentages of students satisfying criteria for these factors (criteria 8, 9, 12, and 15; Fig 1B).

**Fig 2:**
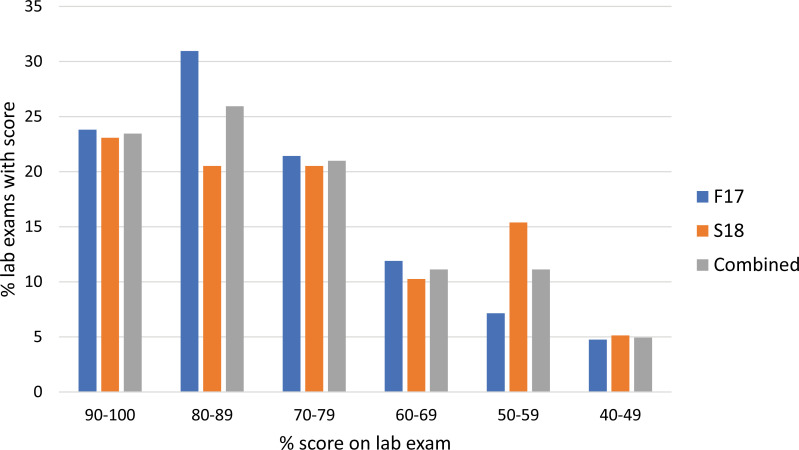
Lab exam scores in the CURE sections. Many students in CURE sections scored high on criteria for experimental design, use of model organisms, and understanding of statistical concepts. Figure represents the percentage of lab reports by the combined total score on 15 questions for 19 total points. Fall 2017, *n* = 42 CURE. Spring 2018, *n* = 39 CURE. “Combined” group represents combined data from Fall 2017 and Spring 2018, *n* = 81 CURE.

The lab exam was scored for 19 total points among 15 questions on experimental design (S7 File). There were eight questions on experimental design (a combination of forced-choice and open-response questions), with a focus on formally understanding the terms “independent variable” and “dependent variable” as they applied to the experiments from class. There was one open-response question on the advantages and disadvantages of model organisms using class examples. There were six questions on statistics (a combination of forced-choice and open-response questions), with a focus on understanding averages, standard deviation, standard error, and statistical significance. From the total scores on the lab exam, 76% of students in Fall 2017 and 64% students in Spring 2018 scored 70% or higher (Fig 2). There was a wider distribution of scores on the lab exam compared to the lab reports, though it was still skewed toward higher scores with 23% of students scoring over 90%, and 26% of students scoring 80–89% in the combined semesters.

The open-response questions on statistics in the lab exam assessed student understanding of fundamental concepts [19]; they were scored for correctly pointing out the basic concept and for providing a correct explanation (S7 and S9 Files). When asked why comparing averages between control and experimental groups is not sufficient to make conclusions, 93% of students pointed out the basic concept, and 57% of students provided a correct explanation of their reasoning (typically by describing how the distribution of values may overlap between the comparison groups). When asked to make judgments on whether a standard deviation is large or not, 70% of students identified the basic concept and 47% provided a correct explanation of the reasoning (typically by describing two contrasting examples where the same standard deviation would be very larger or very small, depending on the mean value).

### Did students in the CURE group show higher levels of research interest, confidence, and scientific mindsets than students in the traditional group?

#### CLASS-Bio assessment

We used the CLASS-Bio instrument to assess changes in student attitudes toward biology (e.g., enjoyment and making real-world connections) and scientific mindsets (e.g., strategies to problem-solving and conceptualizations about the process of knowledge-generation). CLASS-Bio student Pre- and Post-course answers are scored in relation to expert answers to calculate a “% favorable shift”, which is positive when students shift toward more expert-like mindsets and negative when they shift toward more novice-like mindsets [30].

**Fig 3:**
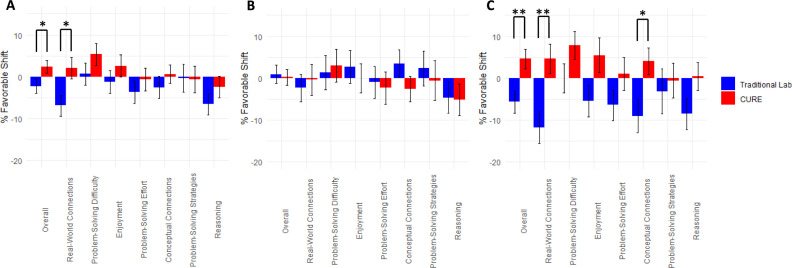
Scientific mindsets on the CLASS-Bio instrument in the CURE and traditional sections. Students in the CURE sections showed more expert-like Pre-to-Post shifts in scientific mindsets compared to students in traditional sections, who mostly experienced shifts toward novice-like mindsets. A. Pre-to-Post shifts in the combined Fall 2017 and Spring 2018 semester; *n* = 83 CURE, *n* = 83 Traditional. B. Pre-to-Post shifts in Fall 2017, *n* = 43 CURE, *n* = 43 Traditional. C. Pre-to-Post shifts in Spring 2018, *n* = 40 CURE, *n* = 40 Traditional.

In overall percentage shifts across questions in all seven categories, students in CURE sections experienced Pre-to-Post shifts toward more expert-like mindsets, while students in traditional sections experienced shifts toward more novice-like mindsets (Fig 3). Compared to traditional students, the CURE students’ overall shift was statistically significant in Spring 2018 and for the combined two semesters, but not in Fall 2017. The difference between the CURE and traditional groups in mean Pre-to-Post shift was 0.31 standard deviation in the combined semesters and was more than double that size (*d* = 0.65) in the Spring semester data. The CURE group showed similar Pre-to-Post shifts on CLASS-Bio relative to all participants in the traditional design including those not in the matched group (S10 File). However, when CLASS-Bio responses for the combined semesters were analyzed as a multivariate response across the seven categories, there was not a significant effect of design (MANOVA *p* = 0.31).

When CLASS-Bio responses are analyzed separately for each of the seven categories, only one of the categories, “real world connection” showed a statistically significant difference in the Pre-to-Post shift between the CURE and traditional groups in the combined data from both semesters (*P* = 0.013). The CURE group showed a relative shift to more expert-like mindsets, while the traditional group did not (Fig 5A). When the two semesters are analyzed separately, none of the categories showed significant differences between the CURE and traditional groups in Fall 2017 (Fig 5B). However, both the “real world connections” and “conceptual connections” categories showed significant pre-post shifts favoring more expert-like mindsets in the CURE group in Spring 2018 (Fig 5C).

#### Research skills assessment

To assess changes in attitudes toward the research process, we compared students in the CURE and traditional sections on measures of student confidence for 14 common research skills, ranging from performing background research to data analysis and presentation. We predicted that CURE students would experience greater gains over the course of the semester compared to traditional students as the CURE design emphasizes a wide range of research skills and student autonomy throughout the process that may improve student confidence. Overall, CURE students reported more positive Pre-to-Post course improvements in research confidence than students in traditional sections (Table 3). CURE versus traditional group differences in Pre-Post gains on the multivariate responses were significant in each semester and in the combined semesters; they did not differ significantly between the two semesters (S10 File). Differences between the traditional and CURE groups explained more than a quarter of the variance in student responses not explained by semester and semester x group effects (partial η^2^ = 0.274). There were statistically significant Pre-to-Post improvement in CURE students in the combined semester data in five areas: 1) developing my own scientific question, 2) designing my own experimental lab protocol, 3) performing statistical analysis, 4) using Excel to make graphs, and 5) writing a lab report (Table 3). Notably, the mean values for confidence on all skills were relatively high on the Pre survey in both groups, with values ranging from 3 to 4.4 on a 5-point Likert scale (S9 File). The two skills that had the lowest Pre-mean scores, “design my own experiment” and “statistical analysis”, were also two of the skills with the greatest improvement in CURE students (Table 3).

**Table 3:** Student confidence in research skills in the CURE and traditional sections.

	Research skills [Post–Pre] changes in means; 5-point Likert scale					
	Fall 2017^1^		Spring 2018^2^		Fall 2017 + Spring 2018^3^	
	Traditional	CURE	Traditional	CURE	Traditional	CURE
1. Work collaboratively and productively in a team	0.21	0.21	-0.17	0.22*	0.03	0.22
2. Perform background research of the scientific literature on a topic	0.07	0.33	-0.2	0.18	-0.06	0.25
3. Critically read the scientific literature on a topic	0.16	0.16	-0.02	0.23	0.08	0.2
4. Develop my own scientific question for an experiment	0.03	0.6**	-0.05	0.6**	-0.02	0.6***
5. Design my own experimental lab protocol	0.02	0.77**	0.05	1***	0.04	0.88***
6. Interpret experimental data (such as finding trends or patterns in data)	-0.12	0.35*	0.25	0.17	0.06	0.26
7. Perform statistical analyses	0	0.72*	0.38	0.55	0.18	0.64*
8. Use Excel to make graphs	-0.02	0.91***	0.23	1.05**	0.09	0.97***
9. Present lab results to my lab members	-0.04	0.28	0.03	0.17	-0.01	0.23
10. Communicate the rationale for doing an experiment to others	0.16	0.25	0.2	0.4	0.18	0.33
11. Discuss a scientific issue by using evidence and developing logical arguments	0.12	0.23	-0.05	0.27	0.03	0.25
12. Write a lab report (with Intro, Methods, Results, Discussion)	0.12	0.53	0	0.6*	0.06	0.57**
13. Write scientifically, but in my own words and avoiding plagiarism	0.21	0.14	0.18	0.52	0.19	0.33
14. Work as an undergraduate research lab assistant in a biology lab	0.07	0.19	-0.02	0.45	0.03	0.31

^1^Fall 2017, *n* = 43 CURE, *n* = 43 Traditional

^2^Spring 2018, *n* = 40 CURE, *n* = 40 Traditional

^3^Fall 2017 and Spring 2018, *n* = 83 CURE, *n* = 83 Traditional

* *P* < 0.05

** *P* < 0.01

*** *P* < 0.001.

#### Student open-response written reflections

**Fig 4:**
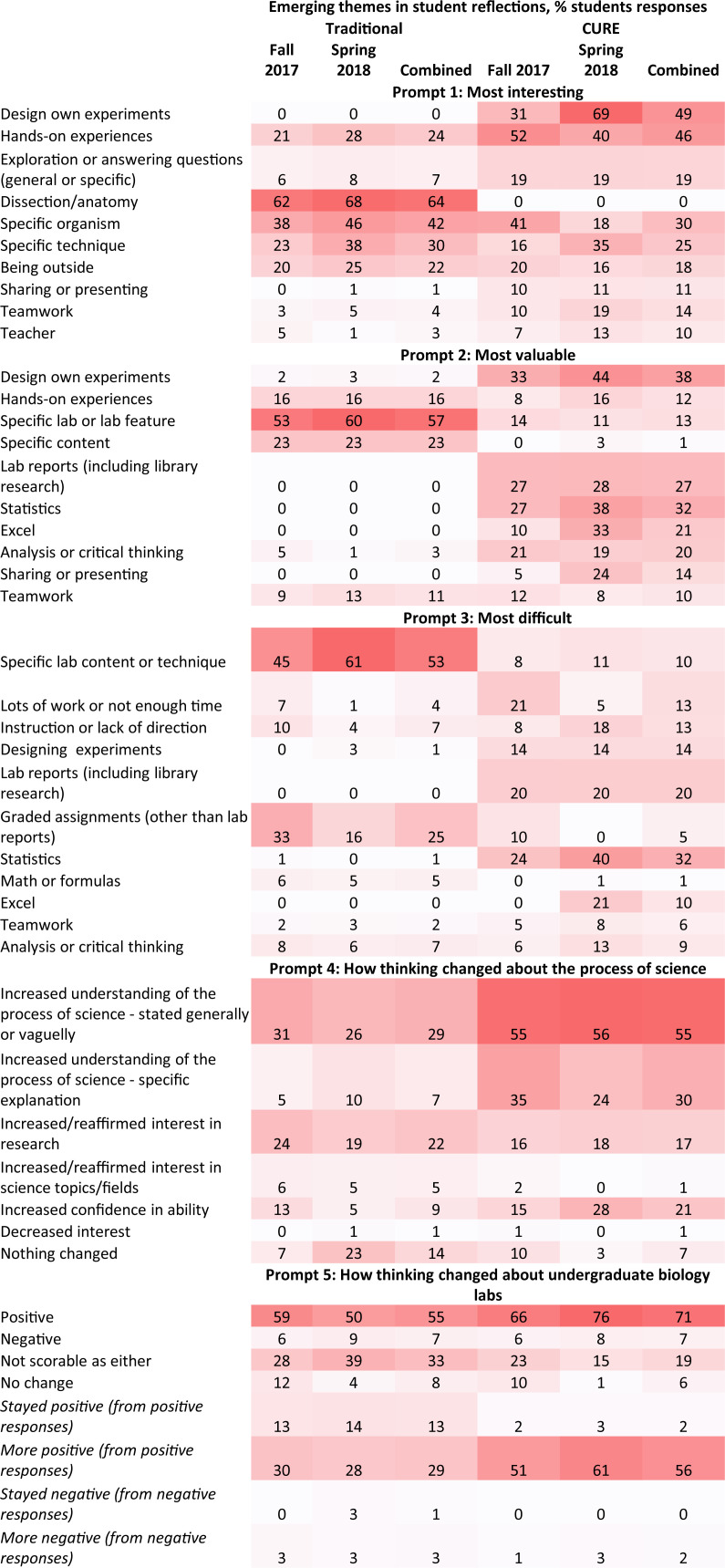
End-of-semester written reflections in the CURE and traditional sections. Open-themed coding of student answers to five prompts revealed different themes in the answers of students in CURE sections compared to students in traditional sections. Fall 2017, *n* = 43 CURE, *n* = 43 Traditional. Spring 2018, *n* = 40 CURE, *n* = 40 Traditional. “Combined” group represents combined data from Fall 2017 and Spring 2018. *n* = 83 CURE, *n* = 83 Traditional. Color intensity is proportional to the percentage of students in each group selecting each response, as shown in each cell.

There were notable differences in the CURE compared to the traditional group on all five prompts, specifically addressing if the course was 1) most interesting, 2) most valuable, 3) most difficult and 4) how thinking has changed about the science process and 5) about undergraduate labs (Fig 4). In the presentation that follows, percentages are listed for the combined two semesters unless otherwise noted. Overall, the traditional group responses were more stable over the two semesters, while there was more variability between the two semesters in the CURE group.

Prompt 1 focused on what aspects of the lab students found most interesting. The most common themes in CURE students’ answers in both semesters were designing their own experiments (49%), hands-on experiences (46%) and specific organisms (30%) (Fig 4). The largest difference among top themes between semesters was for the “designing your own experiments” theme (31% Fall, 69% Spring). The top themes for Prompt 1 in traditional students’ answers were dissections (64%), specific organisms (42%), or specific techniques (30%). Traditional students also described interest in hands-on experiences (24%), albeit at a lower rate than CURE students (46%). Dissections were mentioned by most traditional group students (64%) as a specific example of a hands-on experience. Both groups were interested in labs that involved being outside (22% Traditional, 18% CURE). CURE students more commonly identified additional themes, such as exploration/answering questions (19%), presenting their work (11%), and teamwork (14%) than traditional students (7%, 1%, and 4%, respectively).

Prompt 2 focused on what aspects of the lab students found to be the most valuable. The most common themes in CURE students’ answers were designing their own experiments (38%), lab reports (27%), statistics (32%), Excel (21%), and critical thinking (20%). The largest difference among top themes between semesters was in the mention of Excel as valuable (10% Fall, 33% Spring). The top themes for Prompt 2 in traditional students’ answers were specific lab or lab features (57%), specific content (23%), and hands-on experiences (16%). CURE students also identified hands-on experiences (12%) and specific lab or lab features as being valuable (13%), as well as presenting their work (14%), but not any specific content (1%). A marked difference between the groups was in the value of critical thinking (3% Traditional, 20% CURE). Teamwork was mentioned as being valuable to the same extent (11% Traditional, 10% CURE).

Prompt 3 focused on what aspects of the lab students found to be the most difficult. The most common themes in CURE student responses were statistics (32%), lab reports including those that mentioned background research (20%) and designing their own experiments (14%). The largest differences between semesters was in the mention of Excel as difficult (0% Fall, 21% Spring), statistics as difficult (24% Fall, 40% Spring), and having a lot of work or not enough time (21% Fall, 5% Spring). The top themes for Prompt 3 in traditional students’ answers were specific lab techniques (53%) and graded assignments (25%); students also mentioned math, teamwork and critical thinking. Teamwork and critical thinking were also present in CURE student responses as difficulties at a higher rate than traditional students (2 and 7% respectively Traditional, 6 and 9%, CURE).

Prompt 4 focused on how thinking about the process of science has changed based on their experiences in the lab course. CURE students mention an increased understanding of the process of science at a higher rate than traditional students (55% CURE vs. 30% Traditional). Moreover, a much larger percentage provided specific explanations about how their understanding has changed (30% CURE vs, 7% Traditional). Students in both groups reaffirm their interest in research (17% CURE vs. 22% Traditional), though more CURE students describe an increased confidence in their ability (21% CURE vs. 9% Traditional). Traditional students, especially in the Spring, describe that nothing has changed in their thinking from their experience (7% Traditional Fall, 23% Traditional Spring, 14% Traditional combined; 7% CURE).

Prompt 5 focused on how thinking has changed about undergraduate biology labs based on student experiences in the lab. We scored whether responses displayed a negative or positive affect or reported no change. Most students in both groups conveyed positive feelings, though more so in the CURE sections and especially in the Spring (71% CURE with 66% in Fall and 76% in Spring, 55% Traditional). In the positive category we additionally scored two sub-categories: “stayed positive” and “more positive”. Answers in these sub-categories required including a clear comparison of the current lab course to other labs in the past, to their expectations from before the lab, or to specifically comment on a specific feature or experience from the lab that led them to feel more positive about the lab. From the students that scored in the positive category, many reported having a more positive attitude as a result of the lab. For the “more positive” sub-score, again more CURE students described being more positive compared to traditional students, and again more so in the Spring (56% CURE with 51% in Fall and 61% in Spring, 29% Traditional). In contrast, for the “stayed positive” sub-score, more traditional students described “staying positive” than in the CURE group (13% Traditional, 2% CURE). Few students in both groups displayed negative attitudes (7% Traditional, 7% CURE). Many student responses could not be scored as positive or negative (33% Traditional, 17% CURE). Similar numbers among both groups wrote about not changing in their opinion (8% Traditional, 6% CURE).

### Did students in the CURE group show greater proficiency in achieving program student learning outcomes than students in the traditional group?

#### SRBCI assessment

**Fig 5:**
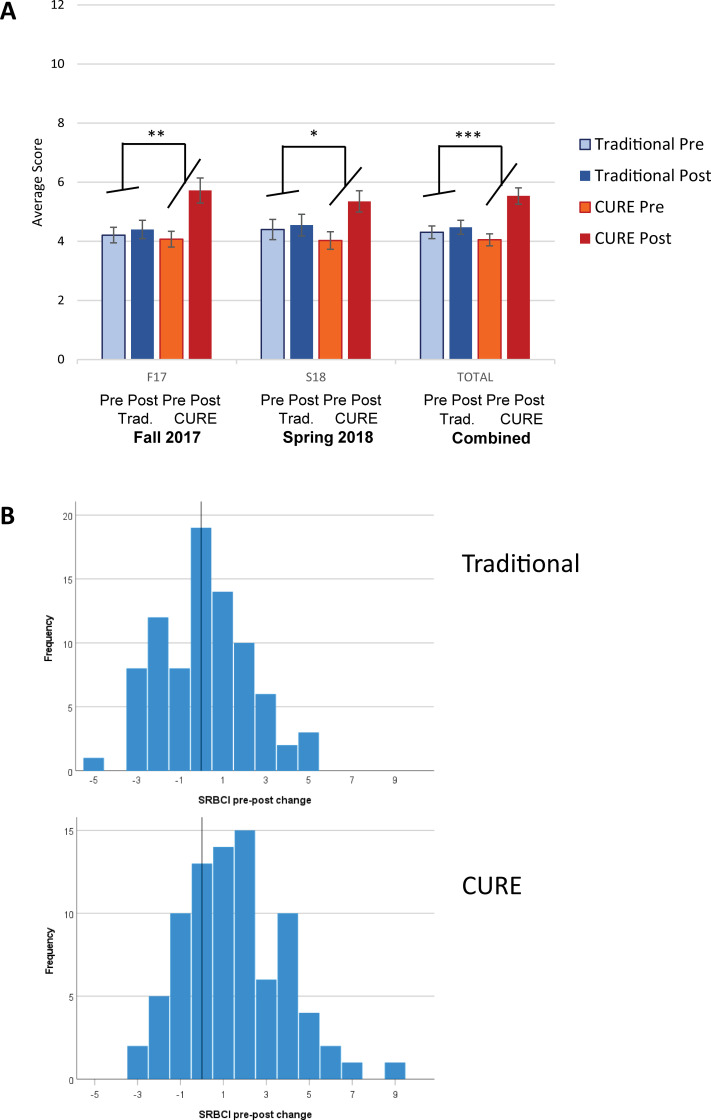
Statistical reasoning ability on the SRBCI instrument in the CURE and traditional curriculum groups. Students in the CURE sections had statistically significant Pre-to-Post improvements in SRBCI scores, but not matched students in the traditional group. SRBCI is a 12-point test. A. Average scores on the SRBCI by group and semester. B. Frequencies of individual Pre-to-Post learning gains by group for the combined semesters. Fall 2017, *n* = 43 CURE, *n* = 43 Traditional. Spring 2018, *n* = 40 CURE, *n* = 40 Traditional. “Combined” group represents combined data from Fall 2017 and Spring 2018. *n* = 83 CURE, *n* = 83 Traditional. The error bars represent the standard error.

We assessed measures of statistical reasoning using the SRBCI to evaluate student ability to apply statistical reasoning to new scenarios that were not the subject of the students’ research in the lab, and thus require students to apply their abilities to new examples without any prior guidance by instructors. The CURE design had instruction in statistical analysis and interpretation, while the traditional laboratory design had no exposure to statistical analysis. Thus, the traditional group served as a comparison to control for learning of statistics outside of BIO 112 laboratory. For statistical reasoning measured by the SRBCI instrument, the Pre-to-Post scores were significantly greater for students in the CURE sections for both semesters and the combined semesters (*p* <0.001 for the combined data; Fig 5A). In the combined data, the larger Pre-to-Post improvement in the CURE group was more than half of the within-group standard deviation of SRBCI scores (*d* = 0.59). Analysis of individual Pre-to-Post learning gains on the SRBCI showed that 64% of CURE group students experienced positive learning gains with a 10-point gain being the highest, compared to 41% for the traditional group students, with a 5-point gain being the highest (Fig 5B). The CURE group similarly showed greater Pre-to-Post improvement on the SRBCI when compared to all participants in the traditional curriculum including those not in the matched group (S10 File).

#### E-EDAT assessment

**Fig 6:**
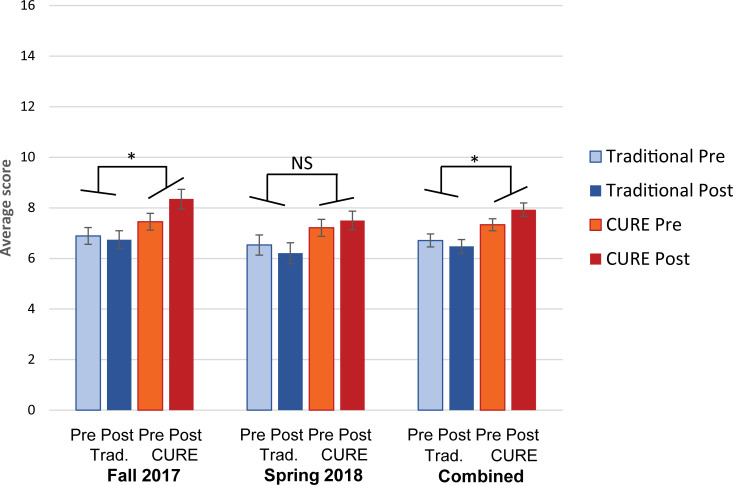
Experimental design ability on the E-EDAT instrument in the CURE and traditional curriculum groups. Students in the CURE sections had statistically significant Pre-to-Post improvements in E-EDAT scores, but not matched students in the traditional sections. E-EDAT is scored on a 17-point rubric. Fall 2017, *n* = 43 CURE, *n* = 41 Traditional. Spring 2018, *n* = 40 CURE, *n* = 39 Traditional. “Combined” group represents combined data from Fall 2017 and Spring 2018. *n* = 83 CURE, *n* = 80 Traditional. The error bars represent the standard error.

We compared CURE and traditional student performance in experimental design using a published instrument that employs an example that was not the subject of instruction. For experimental design measured by the E-EDAT instrument, the net Pre-to-Post improvements were significantly greater for students in the CURE sections compared to the traditional sections in Fall 2017 and in the two combined semesters, but not in Spring 2018 (Fig 6). However, the E-EDAT Pre-test scores were significantly higher overall in the CURE group in both semesters. When semesters were combined, the CURE group had mean Pre-test scores of 0.30 standard deviation higher than the traditional group. In the Post-test, this increased to 0.61 standard deviation. Analysis of individual Pre-to-Post learning gains on the E-EDAT showed that 65% of CURE group students experienced positive learning gains, compared to 35% of traditional groups students (S10 File).

## Discussion

For the scaffolding of the CURE curriculum across the biology major to succeed, introductory students must develop a strong foundation in research skills to build on in the mid- and upper-level courses. As part of the CUR Transformations Project goals at UNCG, the undergraduate biology program SLOs were revised to strengthen the previous emphasis on scientific inference in the curriculum so students complete their undergraduate degree with proficiency in all facets of research inquiry. The new program SLOs specifically address proficiency in research design and hypothesis development, which had been left out of the previous SLOs due to a perception that achieving them was too much to expect for most undergraduates. The Biology CURE curriculum was then designed to provide experience in authentic research in multiple required courses at the introductory and mid-levels and elective courses at the advanced levels.

### Most CURE students met course learning objectives in experimental design and statistical reasoning

The results from the introductory level CURE that we report on here demonstrate that most students were able to achieve learning objectives focused on research proficiency and many students achieved the standards at a high level (57% of students achieved scores of 91% or higher). Most students were also able to achieve the minimum proficiency threshold of scores of 70% or higher on the laboratory exam and a quarter of students achieved scores greater than 90%. Most students showed a general understanding of fundamental concepts in statistics to include averages and standard deviation, and their application to comparison among experimental treatment groups. A smaller but substantial proportion (47–57%) were able to provide clear and specific explanations for their answers, higher-level skills that will need to be developed more fully in more advanced courses through our scaffolding of CURE opportunities.

CURE students demonstrated a strong understanding of their research projects and the elements of controlled experimental design, two areas in which students historically struggle in the traditional laboratory design that uses pre-designed activities. The scoring rubric for the lab report was especially rigorous in the five criteria on evidence-based conclusions. Specifically, student answers were scored as positive only if they both 1) described in the conclusion the statistical analysis, confounding variables, or limitations of the experimental design or execution, and 2) clearly explained how each of these elements impacts the conclusions. Overall, more than 70% of students were able to explain their conclusions on these items.

### CURE students show engagement with and confidence in the research process

Students in CURE sections showed statistically significant improvements in their confidence in five research skills during the two semesters of the study compared to students in traditional sections. Three of the skills that showed differential improvement in CURE students (statistical analysis, graphing using Excel, and lab reports) are typically challenging for students at our institution and are required in upper-level coursework. Two of the skills (develop my own scientific question and design my own experimental lab protocol) also indicate students feel a sense of autonomy when engaging in an authentic research experience. The interpretation of these higher scores reflecting student autonomy is supported by the high prevalence among CURE students of the “design my own experiments” score as being most interesting element of labs in the end-of-semester reflections. The students’ improved confidence in their research skills was supported by the improved student performance on several measures of experimental design and statistical analysis ability.

Research skills have been associated with student interest in research careers, and this effect is partially mediated by student confidence in their ability to successfully engage in scientific inquiry, or self-efficacy [35]. Self-efficacy has been identified as an important factor for STEM student success, and of even greater importance for students who belong to underrepresented groups in STEM disciplines [22–25, 36–38]. Students in both curricular modes started with relatively high mean values of research confidence (3–4.4 out of 5 possible), even in areas where most have had minimal or no prior experiences (e.g., designing their own experimental protocol, statistical analysis, or graphing in Excel). In this manner, the negative Pre-to-Post shift observed especially in the traditional design group can be attributed to a realistic adjustment of skill competency; about half of the students in the study were freshmen and another quarter were sophomores.

CURE students also showed an overall shift toward more expert-like scientific mindsets on the CLASS-Bio instrument, and one that overcame the shift toward more novice-like mindsets in the traditional group. “Real-world connection” is one of the categories that showed a significant improvement in the CURE group compared to the traditional group. This category includes items related to enjoying and finding value in learning biology (e.g., “I enjoy figuring out the answers to biology questions” and “Reasoning skills used to understand biology can be helpful to my everyday life”). “Conceptual connections” is the second category that showed significant improvement in the CURE group in the Spring semester. This category probes an individual’s epistemological disposition on the nature of science (e.g., “For me, biology is primarily about learning known facts as opposed to investigating the unknown” and “I do not expect the rules of biological principles to help my understanding of the ideas”). The improvement in the CURE groups thus likely reflects the overall positive experience and engagement in open-ended investigation.

The analysis of the end-of-semester written reflections supports and expands on the findings from the CLASS-Bio instrument that the CURE group showed improved attitudes and scientific mindsets compared to the traditional group. CURE section student answers generalize about their engagement with the research process throughout the five written reflection prompts, while traditional section student answers are focused on specific labs or content. While both groups describe the labs as a positive experience, many more students in the CURE do so, and a larger proportion specifically describe how the CURE has led them to have a more positive attitude than before. Similarly, while both groups describe that they feel that their understanding of the process of science has increased, many more CURE students provide a specific explanation of how exactly their understanding has improved based on their specific experiences during the CURE lab course.

Students in both groups describe enjoying hands-on activities as one of the top-scoring codes though more CURE students mention hands-on activities. Most students in the traditional group identify the most extensive hands-on experience for that design, animal dissections, as the most interesting to them. CURE students state that designing their own experiments was the most interesting aspect of their lab experience. We included “own” in the code “design own experiments” to accurately reflect how students were emphasizing their autonomy in directing their own research. The high prevalence of this code appearing in student assessments may be indicative of high levels of project ownership that students feel and thus may contribute to improved science identity and sense of belonging. It would be valuable to test this hypothesis more directly in the future, as these characteristics have been linked to student success in STEM degrees and careers [11, 22, 24, 39, 40]. Prior research indicates that the positive effect on undergraduates’ research career intentions in CURE are mediated by student ownership [11].

CURE students additionally describe that the most valuable aspects of the course to them were designing their own experiments, statistics, using Excel, and lab reports. These findings indicate an important alignment between what students find valuable and the curricular goals. Interestingly, CURE students also identify statistics, lab reports, and to a lesser extent designing their own experiments and using Excel as the most difficult. Taken together, these findings indicate that while students found the research process difficult, they found those experiences valuable for their education and appreciated being closely involved with the research process. Perhaps their positive experiences helped them persist through the difficulties, at least in the context of the supportive structure we placed in the context of this CURE where they could work through difficult tasks during the lab.

### CURE students showed higher proficiency in achieving program student learning outcomes

Students in CURE sections had higher Pre-to-Post gains than students in traditional sections on assessment questions about experimental design. The significantly greater gains on the E-EDAT in the CURE group compared to the traditional group could represent gains in experimental design ability, while the greater enthusiasm in the CURE group could also contribute. As evidence for the latter, the pre-E-EDAT mean scores for the CURE students were higher for the CURE students than the traditional students both in Fall 2017 (difference of 0.46) and Spring 2018 (difference of 0.68). These differences are similar in magnitude to the nearly half-point gain on the E-EDAT measured in a previous study after an activity in which students designed an experiment compared to a group that analyzed an experiment [20]. The E-EDAT is an open-ended instrument that requires extensive writing, and the quality and amount of writing is likely influenced by student motivation. The E-EDAT is scored on how many of the 17 elements students have in their answers, so the quality and amount of writing is expected to correlate with the score. The students in both groups had been presented with the syllabus before the first lab session of the semester when the pre-E-EDAT was administered. However, the emphasis in the traditional labs was on the rules of the lab, while in the CURE labs it was on the experiments the students would be designing. Thus, it is possible that the CURE students already had increased enthusiasm for the course, due either to the more positively phrased introduction or anticipation of designing their own experiments.

For the CURE design, we added statistical analysis as part of the expectations for analyzing data and interpreting the results. These concepts were not part of the traditional laboratory design, and thus introduced a higher standard of quantitative reasoning to the learning objectives and student expectations that is more consistent with both the previous and revised program SLOs. Students in the CURE group had significantly higher greater Pre-to-Post gains than students in traditional labs on statistical reasoning as measured by the SRBCI. The differential gains in the CURE group compared to the traditional group demonstrate that students in both groups are not learning statistical reasoning concepts in concurrent classes outside of their BIO 112 laboratory class, as the traditional laboratory students showed no learning gains. The items on the SRBCI emphasize using statistical reasoning to draw conclusions from research data, and not the mechanics of conducting statistical analysis or interpreting formal test statistics. The SRBCI gains in the CURE laboratory student group may reflect teaching of statistical analysis and could also be impacted by teaching in the context of research-based exercises. The differential impact of each of these two factors should be evaluated in further studies.

Student performance on the E-EDAT and SRBCI also suggests areas for future improvement. While CURE section students improved their scores over the course of the semester, the scores are relatively low, though comparable to a similar group of introductory biology students [41]. Purposeful, repeated practice in different contexts in experimental design and statistical analysis targeting areas that have been previously established as difficult [18–21, 42] is expected to result in improved learning. This underscores the importance of departmental reform that scaffolds skills across the curriculum so that students gain experience with research skills multiple times as they progress in their education. Since the administration of the SRBCI is tightly controlled and includes items that have been shown to be difficult [21], it is likely that repeated administration will measure actual learning. Similarly, standardized instruments such as the BEDCI [43] or TIED [44] could be used to track experimental design skills through repeated administration.

### Similar improvements in learning and attitudes in the 3-module and the 2-module CURE modalities

We did not find significant differences in performance on either the E-EDAT or SRBCI for the 3-module CURE in Fall 2017 or the 2-module version in Spring 2018. This could be due to the fact that both modalities involve a CURE design throughout the entire semester, with repetition in the experimentation and in the statistical analysis (statistical analysis was performed for two experiments in both semesters). With regard to attitudinal measures, students in the Spring 2018 semester showed larger shifts on the CLASS-Bio and in some respects in the end-of-semester reflections. Specifically, student enthusiasm was reflected in results that showed most CURE students in the Spring (69%) described their interest in designing their own experiment compared to 31% in the Fall, while the related interest in hands-on experiences remained fairly stable (Fall, 52%, Spring 40%). The 2-module version in the Spring 2018 may allow students to enjoy the experiments more and reduce stress as they have more time for the various steps, and this was our rationale for testing out the reduced module version. This aspect may contribute to the difference in how many students mentioned the lab being a lot of work or not having time, with 21% in the Fall compared to only 5% in the Spring. The work in the lab is significant as students completed their own individual lab report for each lab and two versus three lab reports in a semester can be a big difference. It is important to note that most of the work on the lab reports was completed during lab time.

We developed and studied a CURE involving multiple experiments during the semester, which to our knowledge has not been reported and studies previously with the exception of Indorf et al. 2019 [3] that switched research projects mid-semester. The majority of CURE studies published involve one CURE project over the course of the semester, either as a stand-alone [4] or in combination with other non-CURE activities [45, 46]. Potential benefits of having more than one CURE experiment in a semester include transfer of research skills to multiple contexts, making use of different pedagogical or research strengths across two or more experiments, and engaging student interest. In our experience, developing CURE modules that build on material that was previously part of the traditional design helped considerably with faculty and laboratory preparatory staff buy-in. Our CURE laboratory model may have benefits in a number of different institutional contexts and can provide instructors and departments with additional choices to consider as they contemplate introductory course redesign.

### The role of motivation and challenge in student success

The findings that students enjoyed, valued, and showed significant learning in a research-based course that they find challenging suggests that reform efforts of implementing CURE into introductory biology curriculum can serve as a vehicle for learning difficult concepts when students are engaged as active agents in the process. Student motivation is an essential component given the recognized importance of student science identity and sense of belonging for STEM success, especially for minority students [22–24, 39, 40]. In fact, experiencing challenges and even failure during the research process when there is an opportunity for iteration in a CURE, as was the case in our study, has been linked to positive changes in student ability to persist with scientific obstacles [12, 47]. When combined with the alignment between student and curricular goals, these metrics also indicate student buy-in for the CURE curricular reform [48].

Our work supports the growing literature on the effectiveness of incorporating challenging, research-based experiences at the introductory level. This study validates the use of research-rich, introductory-level laboratory curricula in a large-enrollment class at a mid-level minority serving public university. This is one of a limited number of studies that report measurement of student outcomes in a non-volunteer population using a controlled design with a comparison group [3, 4, 11, 13, 45, 49]. Students in both groups demonstrate that they value hands-on experiences and have a positive attitude toward laboratory courses. However, students in the CURE sections show greater gains in learning, enthusiasm, appreciation and confidence, despite the self-reported difficulty of the course and the inclusion of learning outcomes previously considered too challenging.

## Supporting information

S1 File Traditional and CURE schedules. (DOCX)

S2 File CURE syllabus. (DOCX)

S3 File Surveys. (DOCX)

S4 File Lab report. (DOCX)

S5 File Lab exam. (DOCX)

S6 File Lab report coding. (DOCX)

S7 File Lab exam coding. (DOCX)

S8 File E-EDAT coding. (DOCX)

S9 File Data. (XLSX)

S10 File Statistical analyses. (DOCX)
