# Supplementary material for: An introductory biology research-rich laboratory course shows improvements in students’ research skills, confidence, and attitudes
Source: PLoS One. 2021 Dec 16;16(12):e0261278. doi: 10.1371/journal.pone.0261278 (PMC8675740; doi:10.1371/journal.pone.0261278)
Supplement: S1 File — (DOCX) [file pone.0261278.s001.docx]

**The Traditional and CURE curricula week-by-week schedule of labs and research elements in each lab**

Research elements

1-Background research or observations/experimentation that lead to steps 2 and 3

1A Background research

1B Observations/experimentation

2-Develop hypothesis

3-Design an experiment

3A Choice of variables

3B Control or comparison groups

3C Design experimental protocol

4-Perform experiment

5-Data collection and recording

6-Data analysis and interpretation

6A non-statistical

6B statistical instruction and activities

6C descriptive statistics

6D inferential statistics

7-Data presentation

7A written

7B oral

| **Week** | **Traditional laboratory** | **CURE Fall 2017** | **CURE Spring 2018** |
| --- | --- | --- | --- |
| 1 | Introduction | Invertebrate diversity I  (1B, 2, 3A/B) | Introduction  (1A,2) |
| 2 | Discovery Science: Using Tools of Biology (observations) | Invertebrate diversity II  (1A, 2, 3A/B, 4, 5) | Caterpillar behavior I  (1A/B,2, 3A/B/C) |
| 3 | Introduction to the Peabody Park Ecosystem  (2,4,5,6) | Invertebrate diversity III  (4,5,6A) | Caterpillar behavior II  (1A,3A/B/C,4,5) |
| 4 | Population Genetics  (2,4,5,6) | Invertebrate diversity IV  (6B/C/D, 7A/B) | Caterpillar behavior III  (1A,3A, 3B, 3C,6B/C/D) |
| 5 | Evolutionary Mechanisms  (2,4,5,6) | Caterpillar behavior I  (1A/B, 3A) | Caterpillar behavior IV  (4,5,6A/B/C/D,7A) |
| 6 | Lab Exam 1 | Caterpillar behavior II  (1A, 3A/B/C) | Caterpillar behavior V  (6A/B/C/D,7A) |
| 7 | Population Genetics and Biotechnology  (4,5,6) | Caterpillar behavior III  (3A/B/C, 4, 5, 7A) | Caterpillar behavior VI  (7,8A) |
| 8 | Evolutionary Relationships  (2,4,5,6) | Caterpillar behavior IV  (6A/B/C/D, 7A/B) | Invertebrate diversity I  (1A/B, 2, 3A/B) |
| 9 | Bacteria and the Evolution of Eukaryotes  (2,3A,4,5,6) | *Daphnia* heart rate studies I  (3A/B/C x2, 4, 5) | Invertebrate diversity II  (4, 5, 7A) |
| 10 | Plant Diversity  (observations) | *Daphnia* heart rate studies II  (3A,4,5,6A/B/C/D) | Invertebrate diversity III  (4, 5, 6A/B/C/D, 7A) |
| 11 | Homeostasis and Animal Diversity  (observations/dissections) | *Daphnia* heart rate studies III  (1A,4,5,6A/B/C/D,7A) | Invertebrate diversity IV  (7A/B) |
| 12 | Lab Exam 2 | *Daphnia* heart rate studies IV  (7B) | Presentations (Invertebrate experiment) and lab exam  (7B) |
| 13 | Make-up week | Presentations (*Daphnia* experiment) and lab exam  (7B) | Make-up week |
