# Supplementary material for: An introductory biology research-rich laboratory course shows improvements in students’ research skills, confidence, and attitudes
Source: PLoS One. 2021 Dec 16;16(12):e0261278. doi: 10.1371/journal.pone.0261278 (PMC8675740; doi:10.1371/journal.pone.0261278)
Supplement: S2 File — (DOCX) [file pone.0261278.s002.docx]

**CURE Syllabus - Fall 2017**

Section: ____ Instructor: _____________________________ Email: _________________________________

Office: _____________ Phone: _____________________ Office Hours: ________________________________

***Please note specifics of what happens in each lab may change. We will notify you via Canvas of all changes.***

The theme for our lab section this semester involves the physiological, ecological, and evolutionary aspects of organismal interaction. More specifically, we will investigate the coevolution of herbivore-plant interactions. Within this theme, you will have the opportunity to:

- Work with live organisms, ask questions about their behavior, and think about the benefits and disadvantages of different model organisms for biological investigations.
- Learn about the web of complex and mutually dependent interactions that connect living things on Earth, including within food webs.
- Think about how the genetics and physiology of living organisms impacts their ecological interactions and evolution.
- Explore the impact of human species on living organisms and propose conservation plans.

[placeholder for relevant exciting images]

In lab you will be performing biological experiments – including those of your own design – and will have the opportunity to repeat and improve your experiments, just like scientists do in reality! In the process, you will be practicing important experimental skills that prepare you to think like a scientist, and to be successful in your subsequent coursework and careers. Specifically, you will:

1. Perform background research and develop hypotheses.
2. Design experiments.
3. Learn and practice laboratory skills.
4. Record and analyze quantitative data, including gaining proficiency in using Excel.
5. Perform statistical significance testing.
6. Evaluate experimental results to suggest improvements to the experimental design or to answer further questions stemming from the results.
7. Perform improved experiments or further exploration based on evaluation of first experiment (as described in 6 above).

**Overview of laboratory experiments**

***You will be collaborating as part of a group of 2-4 students to decide on and perform your experiments***

| **Lab** | **Description** |
| --- | --- |
| **Lab #1.**  What’s lurking under foot at UNCG? Exploring the amazing world of soil and leaf litter. | Have you ever wondered why some ecosystems have more numbers and types of organisms than others? In lab #1, you will explore the biodiversity of our campus by comparing the numbers and types of soil invertebrates in two habitats of your choosing. You will use Berlese funnels, simple classification keys and microscopes to catalogue the myriad of tiny creatures that live beneath your feet. Then, you will compare these data with observations and measurements about the two habitats, and share your results with the class. Maybe you will discover a link between habitat characteristics and biodiversity!  Lab #1 will introduce you to experimental design, data collection, graphing in Excel, and statistics, and you will practice scientific writing in the form of a guided mini lab report. |
| **Lab #2.**  Caterpillar food preference behaviors | In the second lab, you will have the opportunity to develop and test your own hypothesis as to what compounds in caterpillar food may determine the food preferences of these notoriously voracious herbivores. How will you know that they are responding to what you’re interested in, and not something else?  You will share your results with the class as before, and will “graduate” to writing a full lab report that will still be guided. The experiments in lab #2 will help you practice your skills in experimental design, data collection, graphing in Excel, and statistics. |
| **Lab #3.**  Effect of plant compounds on Daphnia physiology | Now that you know that many plant compounds (caffeine! aspirin!) have strong effects on animals, you will have the chance to study directly for yourself their effect on animal physiology. Lab #3 also provides you with the opportunity to participate more fully in the real process of science, which involves background research and repeating experiments in order to improve upon them.  First, you will design and perform an experiment to test the effect of caffeine or ethanol on Daphnia heart rate. After class discussion and further deliberation, you will have an opportunity to improve your experiment. Based on what you learned in the first two lab sessions, you will research and test a plant substance of your own choice.  You will again write-up your experiment in the form of a guided full lab report. This time, you will also present the results of your experiment in the form of a PowerPoint presentation to the class. The experiments in lab #3 will help you solidify your skills in experimental design, data collection, graphing in Excel, and statistics. |

**Summary of lab components**

***The lab grade represents 25% of your total grade for 112***

| **Lab component** | **Contribution to grade** |
| --- | --- |
| Active group and lab participation | 5% |
| Homework & in-class activities | 30% |
| Guided lab reports | 40% (*1 x 10%, 2 x 15%)* |
| Shares & presentation | 20% *(2 x 5%, 1 x 10%)* |
| End-of-class final assessment | 5% |

- There will be time in lab to work on the write-ups. Most assignments are posted electronically to Canvas.
- **Let us know AS EARLY/AS SOON AS POSSIBLE if there is any planned or unplanned event in your life that impacts this course. Do not hesitate to email your instructor IMMEDIATELY with any issues.**

**Tentative Lab & Task Schedule**

| **Week** | **Lab #** | **Lab** | **Tasks for you to complete** |
| --- | --- | --- | --- |
| 1  **Aug. 16/17** | 1 | Biodiversity I | - Intro to topic and course. Start-of-class Survey. - Peabody Park field trip #1: Intro & observations. - *HW: Effect of non-native plants on herbivore diversity reading.* |
| 2  **Aug. 23/24** | 2 | Biodiversity II | - Peabody Park field trip #2: Data collection; set up Berlese funnels - Data recording: Intro to Excel. - Lab write-up: part I. - *HW: MathBench graphing activity & identification activity.* |
| 3  **Aug. 30/31** | 3 | Biodiversity III | - Extract and identify soil invertebrates. - Data recording: Intro to graphing in Excel. - Lab write-up: part II. - *HW: Shannon biodiversity index activity.* |
| 4  **Sep. 6/7** | 4 | Biodiversity IV | - Statistics mini-lecture and activity. - Data analysis: Chi-square stats in Excel. Shannon diversity index. - *Biodiversity lab Share.* - *HW: “Chemical warfare” reading.* |
| 5  **Sep. 13/14** | 5 | Caterpillar I | - “Chemical warfare” reading – discussion - Background literature research: Intro to sources. - Caterpillar student choice experiment – background research & observations/measurements of caterpillar larvae. - *Biodiversity Lab Report due.* |
| 6  **Sep. 20/21** | 6 | Caterpillar II | - Caterpillar student choice experiment – observations/measurements of caterpillar larvae & design own experiment. - Experimental design: Intro to controlled experiments. |
| 7  **Sep. 27/28** | 7 | Caterpillar III | - Caterpillar student choice experiment – set up. - Data analysis: Energy dynamics calculations. |
| 8  **Oct. 4/5** | 8 | Caterpillar IV | - Caterpillar student choice experiment – results & analysis, write-up. - *Caterpillar lab Share.* |
|  |  |  | FALL BREAK*.* NO 112 LABS |
| 10  **Oct. 18/19** | 9 | Daphnia I | - Daphnia lab: design & perform experiment. - Experimental design: Improving experiments. - Daphnia lab: Re-design own experiment. - *Caterpillar Lab Report due.* |
| 11  **Oct. 25/26** | 10 | Daphnia II | - Daphnia lab: perform re-designed experiment. - Daphnia student choice experiment – background research, design. |
| 12  **Nov. 1/2** | 11 | Daphnia III | - Daphnia student choice experiment – perform experiment. - Daphnia lab: write-up & prepare for presentations. |
| 13  **Nov. 8/9** | 12 | Daphnia IV | - *Daphnia lab Presentations: part I.* - Review session for Final Assessment on exp. design & statistics - End-of-class Reflection & Feedback. - *Daphnia Lab Report due.* |
| 14  **Nov. 15/16** | 13 | *Grand Finale* | - *Daphnia lab Presentations: part II.* - End-of-class Final Assessment on exp. design & statistics |

*”**Aug 15/16”** - the first date is the section 12 date, the second date is the date for sections 13 & 14.

**Course Policies**

**Course Materials & Access:**

You do NOT need to buy the 112 Lab Manual.  You will need a 1.5” 3-ring binder and hole-punched blank paper for writing notes and ideas. We use computers in every lab session to access assignments, and to record and analyze data. We have a limited number of laptops available in lab; if you have one, please bring your own laptop or tablet to each lab. All other materials will be provided.

In many cases, we will use Canvas to distribute handouts and collect assignments. Please call 336-256-8324 (6-TECH) if you are having difficulty with your accounts. To make sure that you receive important announcements and emails, log into Canvas, click on Account, and go into Settings to add a preferred email address or phone number. Also under Account, go into Notifications to indicate how you would like to be contacted and how often. I will be emailing you through Canvas for the remainder of the semester, so make sure to check your Canvas email daily, or have it forwarded to an email you do check regularly.

**Safety & Dress:**

It is not safe to put anything in your mouth while in a lab room so we must insist on no food, drink, gum, candy, lip gloss, etc. during your laboratory experience. Please wear closed toe shoes to protect your feet, and sturdy shoes that you don’t mind getting dirty on days when there are field trips. Science is messy so please dress with that in mind. Be mindful in following any safety instructions given during lab and be sure to use the safety equipment we provide when it is needed. *Please discuss with your instructor if you have any medical or safety concerns.*

**Engaged Participation:**

Because this laboratory experience requires authentic collaboration, you must be actively engaged in this course. This means arriving on time, and being prepared to learn, contribute, discuss, and problem solve with your team. You must be an active listener and solicit input from all members of your team as well as being ready to contribute your own ideas and opinions. If you are late or absent, you should be prepared to apologize to your team, explain to your instructor, and potentially take a deduction in your engagement grade. *Always contact you instructor as soon as possible if you are going to be absent or tardy, or if you have any problems or concerns.*

**Academic Integrity:**

In this course we expect you to work together in designing and carrying out experiments, including data collection. We expect data analysis (e.g., calculations, graphing, and statistics) and writing to be done individually, on your own. Unless we tell you otherwise on a specific assignment, signing the Academic Integrity Policy is your pledge that you have followed by these guidelines. Please read about the Integrity Policy here. *If you have any questions or difficulties when you’re working on your own or in a group, just consult with your instructor. Do not hesitate to ask for help.*

**Problems, Issues, Concerns, and Questions**:

Learning is a process that takes place outside of the realm of the familiar or comfortable. We hope this learning experience will be challenging, exciting, stimulating and fun but we know that at times you might find it hard and frustrating. Please be your own best advocate for learning by bringing your problems, issues, concerns, and questions to your instructors as they arise. *We are here to help. Just ask!*

[placeholder for relevant exciting images]

**CURE Syllabus – Spring 2018**

Section: ____ Instructor: _____________________________ Email: _________________________________

Office: _____________ Phone: _____________________ Office Hours: ________________________________

***Please note specifics of what happens in each lab may change. We will notify you via Canvas of all changes.***

The theme for our lab section this semester involves the physiological, ecological, and evolutionary aspects of organismal interaction. More specifically, we will investigate the coevolution of herbivore-plant interactions. Within this theme, you will have the opportunity to:

- Work with live organisms, ask questions about their behavior, and think about the benefits and disadvantages of different model organisms for biological investigations.
- Learn about the web of complex and mutually dependent interactions that connect living things on Earth, including within food webs.
- Think about how the genetics and physiology of living organisms impacts their ecological interactions and evolution.
- Explore the impact of human species on living organisms and propose conservation plans.

[placeholder for relevant exciting images]

In lab, you will be performing biological experiments – including those of your own design – and will have the opportunity to repeat and improve your experiments, like scientists do in reality. In the process, you will be practicing important skills that prepare you to think like a scientist, and to be successful in your subsequent coursework and careers. Specifically, you will:

1. Learn and practice laboratory skills.
2. Perform background research, develop hypotheses, and design experiments.
3. Record, analyze, and display quantitative data, including gaining proficiency in using Excel for graphing and statistical significance testing.
4. Evaluate experimental results to suggest improvements to the experimental design or to answer further questions stemming from the results and perform improved experiments or further exploration based on evaluation of first experiment.

**Overview of laboratory experiments**

***You will be collaborating in groups of 2-4 students to design and perform your experiments.***

| **Lab** | **Description** |
| --- | --- |
| **Lab #1.**  Caterpillar food preference behaviors | In the first lab, you will have the opportunity to develop and test your own hypothesis regarding compounds that may determine the food preferences of these notoriously voracious herbivores. In the process, you will examine confounding factors to answer the question, how will you know that the caterpillars are responding to the compound, and not to something else?  Lab #1 will introduce you to experimental design, data collection, graphing in Excel, and statistics. You will share your results with the class and practice scientific writing in the form of a guided lab report. |
| **Lab #2.**  What’s lurking under foot at UNCG? Exploring the amazing world of leaf litter. | Have you ever wondered why some ecosystems have more numbers and types of organisms than others? In lab #2, you will explore the biodiversity of our campus by comparing the numbers and types of soil invertebrates in two habitats of your choosing. You will use Berlese funnels, simple classification keys and microscopes to catalogue the myriad of tiny creatures that live beneath your feet. Then, you will compare these data with observations and measurements about the two habitats, and share your results with the class. Maybe you will discover a link between habitat characteristics and biodiversity!  You will share your results with the class, and will write another guided lab report. The experiments in lab #2 will help you practice your skills in experimental design, data collection, graphing in Excel, and statistics. |

**Course Policies**

**Course Materials & Access:**

You do NOT need to buy the 112 Lab Manual.  You will need a 1.5” 3-ring binder and hole-punched blank paper for writing notes and ideas. We use computers in every lab session to access assignments, and to record and analyze data. We have a limited number of laptops available in lab; if you have one, please bring your own laptop or tablet to each lab. All other materials will be provided.

You must have access to desktop versions of microsoft word, excel and powerpoint in order to complete assignments and activities. You may download microsoft office for free at <https://its.uncg.edu/office365/>. Be sure you have microsoft office installed on your laptop before Lab 2.

In many cases, we will use Canvas to distribute handouts and collect assignments. Please call 336-256-8324 (6-TECH) if you are having difficulty with your accounts. To make sure that you receive important announcements and emails, log into Canvas, click on Account, and go into Settings to add a preferred email address or phone number. Also under Account, go into Notifications to indicate how you would like to be contacted and how often. I will be emailing you through Canvas for the remainder of the semester, so make sure to check your Canvas email daily, or have it forwarded to an email you do check regularly.

**Safety & Dress:** It is not safe to put anything in your mouth while in a lab room so we must insist on no food, drink, gum, candy, lip gloss, etc. during your laboratory experience. Please wear closed toe shoes to protect your feet, and sturdy shoes that you don’t mind getting dirty on days when there are field trips. Science is messy so please dress with that in mind. Be mindful in following any safety instructions given during lab and be sure to use the safety equipment we provide when it is needed. *Please discuss with your instructor if you have any medical or safety concerns.*

**Engaged Participation, Absences, and Tardiness:**

Because this laboratory experience requires authentic collaboration, you must be actively engaged in this course. This means arriving on time, and being prepared to learn, contribute, discuss, and problem solve with your team. You must be an active listener and solicit input from all members of your team as well as being ready to contribute your own ideas and opinions.

Off task use of electronics is prohibited and will result in deductions in your participation grade.

If you are not in your seat and ready to contribute at the beginning of lab, you should be prepared to apologize to your team, explain to your instructor, and take a deduction in your engagement grade.

If you know you will be absent due to illness for which you have received medical attention, death in the family, or participation in UNCG activities like conference or competitions, you must contact your instructor ASAP. Otherwise, your absence is considered unexcused. Your lab grade will be lowered by 10% for each unexcused absence. In addition, it may not be possible to make-up work done in lab and if your absence is unexcused, you may also receive a zero for missed work. *Always contact you instructor as soon as possible if you are going to be absent, or if you have any problems or concerns regarding attendance.*

**Academic Integrity:**

In this course we expect you to work together in designing and carrying out experiments, including data collection. We expect data analysis (e.g., calculations, graphing, and statistics) and writing to be done individually, on your own. Unless we tell you otherwise on a specific assignment, by submitting the assignment, you pledge that you have followed by these guidelines. Please read about the Integrity Policy here: <http://sa.uncg.edu/dean/academic-integrity/> and see additional information regarding group vs individual work in the attached handout, “Independent vs. Group Work in Bio 112L.” *If you have any questions or difficulties when you’re working on your own or in a group, just consult with your instructor. Don’t hesitate to ask for help.*

**Assignments & Grading:**

Assignments will be posted on Canvas and/or given out during lab. All due dates will be clearly stated and unless you can document extenuating circumstances, late work will be penalized 10% per day, starting with the day it was due. You will have time to work on some assignments during lab. If you are unable to complete an assignment that is due by the end of lab, it is your responsibility to discuss an extension with your instructor.

Your lab grade represents 25% of your total grade for 112. You must pass both lab and lecture in order to receive a passing grade in the course.

**Summary of lab components**

| **Lab component** | **Contribution to grade** |
| --- | --- |
| Engaged participation | 10% |
| Homework & in-class activities | 35% |
| Guided lab reports | 30% (*2 x 15%)* |
| Shares & presentation | 20% *(2 x 10%)* |
| End-of-class final assessment | 5% |

**Problems, Issues, Concerns, and Questions**:

Learning is a process that takes place outside of the realm of the familiar or comfortable. We hope this learning experience will be challenging, exciting, stimulating and fun but we know that at times you might find it hard and frustrating. Please be your own best advocate for learning by bringing your problems, issues, concerns, and questions to your instructors as they arise. *We are here to help. Just ask!*

**Let your instructor know AS EARLY/AS SOON AS POSSIBLE if there is any planned or unplanned event in your life that impacts this course. Do not hesitate to email your instructor IMMEDIATELY with any issues.**

**Preliminary lab and task schedule Spring 2018**

| ***Week of:*** | ***Lab #*** | ***Lab*** | ***Tasks for you to complete*** |
| --- | --- | --- | --- |
| Jan 8-12 | 0 | Intro | - Introduction to course - Caterpillar lab challenge - Introduce Chemical Warfare paper - *Start-of-class survey* |
| Jan 15-19 |  | MLK Week | No labs this week |
| Jan 22-26 | 1 | Caterpillar 1 | - Caterpillar lab challenge - ”Chemical warfare” reading -discussion - Observations and design behavioral experiment. Lab decides on common protocol - *HW 1.1 Chemical Warfare Reading* |
| Jan 29-Feb 2 | 2 | Caterpillar 2 | - Run Class Caterpillar Experiment 1:Food preference - Intro to graphing in Excel - Background literature research: Intro to sources - *HW 2.2 MathBench Graphing Activity* |
| Feb 5-9 | 3 | Caterpillar 3 | - Intro to Statistics: Sampling & Chi-Square - Analyze data food preference data and draw conclusions - Experimental design: intro to controlled experiments - Choose compound to test and design feeding & growth experiment - *HW 1.3 MathBench Normal Distribution Activity* |
| Feb 12-16 | 4 | Caterpillar 4 | - Set up Student Choice Caterpillar Experiment 2: Feeding & Growth - Write up introduction & methods - Intro to Statistics: Distribution and T-test - *HW 1.4 MathBench Standard Error* |
| Feb 19-22 | 5 | Caterpillar 5 | - Energy dynamics calculations - Student Choice Caterpillar Experiment 2: Feeding & Growth data collection, results & analysis write-up |
| Feb 26-Mar 2 | 6 | Caterpillar 6 | - Work on Presentations & Lab Report - *Caterpillar Presentations* - *Caterpillar Lab Report Due March 4* |
| Mar 5-9 |  | Spring Break | No labs this week |
| Mar 12-16 | 7 | Diversity 1 | - Diversity Lab challenge - Peabody Park field trip #1: Intro and observations, choose sites to sample, collect abiotic factors data - *HW 2.1 What makes one habitat more diverse than another?* - *HW 2.1 Non-native Plants and Herbivore Diversity Reading* |
| Mar 19-23 | 8 | Diversity 2 | - Peabody Park Field trip #2: setup Berlese funnels - Identifying Soil Invertebrates mini-lecture - Practice identifying soil invertebrates - *HW TBA* |
| Mar 26-29 | 9 | Diversity 3 | - Extract and identify soil invertebrates - Enter data in Excel - *HW TBA* |
| Apr 2-6 |  | Winter Break | No labs this week |
| Apr 9-12 | 10 | Diversity 4 | - Graph diversity data in Excel - Chi-square stats in Excel, Shannon diversity index - Work on Biodiversity presentations and lab reports - *HW TBA* |
| Apr 16-19 | 11 | Grand Finale | - *Biodiversity presentations* - *Biodiversity Lab Report Due* - End-of-class survey - End-of-class final assessment on experimental design and statistics |

**Let your instructor know AS EARLY/AS SOON AS POSSIBLE if there is any planned or unplanned event in your life that impacts this course. Do not hesitate to email your instructor IMMEDIATELY with any issues.**

**Independent vs. Group Work in Bio 112L**

Independent work doesn’t mean that you work in a vacuum and group work doesn’t mean that you are not contributing actively. Here is some additional guidance on group vs. independent work in BIO 112L.

**Group Work**

- Planning the experiment
- Doing the experiment (e.g., collecting specimens, making measurements)
- Making the data table without calculations
- Preparing the group presentation (e.g., the file, the oral script)

Group work means that you are actively participating – intellectually or physically – with the task at hand. That you are an active contributor, and are helping the group achieve better results than if each person was on their own. Much work in our human society is group work. Working in a group has it challenges, and it requires practice to navigate between your individual characteristics and needs and those of your partner and the group as a whole. Since our lab has ample opportunity for group work, this semester is a good time and place to work on improving your groups skills. Challenge yourself to work through the more difficult parts of group work. For example:

- Do you just take the first suggestion or explanation that a group partner gives? Are you able, instead, to help improve a suggestion or explanation? To suggest your own? To speak up for your ideas or needs?
- When you are moving more quickly or slowly than your group, what do you do? Are you able to negotiate your own learning space, requesting time to process on your own, asking questions, or using your knowledge to guide someone through the process without doing it for them?
- Are you providing leadership to your group? Or do you complete your own work and the leave your group members to struggle on their own? Do you come as prepared as possible, having completed the homework thoughtfully and to the best of your ability? Are you being the team member you would want to have on your team if you were either the most or least competent at the task at hand?

**Independent work**

- Performing any calculations
- Making a graph
- Performing statistical analysis
- Writing introduction for lab report
- Writing the discussion for lab report

Independent work means that *you* are going through the process *yourself* in a way that leads *you* to be able to talk or write about something correctly or to do something *yourself* – for example, that *you* can make a graph using Excel. The objective is that YOU have the opportunity to engage in a process that leads YOU to LEARN.

Independent work does not mean that you cannot get help as you are going through the learning process. For example, if you’re looking for the “column graph” button in Excel and can’t seem to find it, you can ask your lab partner or the instructor. The point is that *you* are making the graph. Same holds true for any calculations.

Of course, you could be getting so much help that at the end you have not done the graph yourself – and will have a difficult time making a second graph on your own that fits the requirements. If you find you need a lot of help – and that is perfectly okay, we’re doing this precisely so that you get the chance to learn – then it is best to turn to the instructor. The instructor has the experience to help you learn – versus just doing it for you.
