# Supplementary material for: An introductory biology research-rich laboratory course shows improvements in students’ research skills, confidence, and attitudes
Source: PLoS One. 2021 Dec 16;16(12):e0261278. doi: 10.1371/journal.pone.0261278 (PMC8675740; doi:10.1371/journal.pone.0261278)
Supplement: S3 File — (DOCX) [file pone.0261278.s003.docx]

**Pre-Survey**

**Part I. General information**

**1) Demographic questions**

1. What is your first name?

2. What is your last name?

3. What is your intended or declared major? You can choose all that apply.

- Biology, Biochemistry, Environmental Studies, Nursing, Pre-health (Premed/Pre-dental/Pre-pharm/etc.), Psychology, Nutrition, Other (please specify)

4.What year is this for your college career?

- 1^st^ year, 2^nd^ year, 3^rd^ year, 4^th^ year, Transfer or returning student, Post-bac, Other

5. What is your age?

- 17 or younger, 18-19, 20-21, 22-23, 24 or older

6. What is your gender?

- Female, Male, Other/do not wish to disclose

**Part II. Biology lab tasks.**

**2) Research skills questionnaire.**

*Likert scale with Not confident, Somewhat confident, Moderately confident, Very confident options.*

How confident do you feel in your ability to perform the following biology lab-based tasks?

1. Work collaboratively and productively in a team.

2. Perform background research of the scientific literature on a topic.

3. Critically read the scientific literature on a topic.

4. Develop my own scientific question for an experiment.

5. Design my own experimental lab protocol.

6. Interpret experimental data (such as finding trends or patterns in data).

7. Perform statistical analyses.

8. Use Excel to make graphs.

9. Present lab results to my lab members.

10. Communicate the rationale for doing an experiment to others.

11. Discuss a scientific issue by using evidence and developing logical arguments.

12. Write a lab report (with Intro, Methods, Results, Discussion).

13. Write scientifically, but in my own words and avoiding plagiarism.

14. Work as an undergraduate research lab assistant in a biology lab.

**Part III. Learning biology.**

**4) BioCLASS instrument**

**Part IV. Experiments.**

**5) E-EDAT instrument**

**Part V. Statistics in experiments.**

**6) SRBCI instrument**

**Post-Survey**

**Part I. End-of-semester reflection.**

**1) Written reflection prompts.**

For the questions below, think back on your whole experience in 112 Lab this semester and describe 2-3 of your top considerations.

1. The aspects of the 112 Labs that were most interesting or fun for me were...

2. The aspects of the 112 Labs that were most valuable for me as a student and/or my future career were...

3. The aspects of the 112 Labs that were most difficult were...

4. I believe that future labs would most benefit from...

5. Overall, what do you think changed the most about how you think and feel about the process of science - experiments, scientific research?

6. Overall, what do you think changed the most about how you think and feel about undergraduate science labs?

7. Space for any other comments that can help us promote the success of future students and education at UNCG.

Thank you for your thoughtful reflection! We hope that it helped you take the time to reflect and plan the next steps in your life.

**2) Research skills questionnaire (see Pre-Survey).**

**Part II. Learning biology.**

**3) BioCLASS instrument**

**Part III. Experiments.**

**4) E-EDAT instrument**

**Part IV. Statistics in experiments.**

**5) SRBCI instrument**
