# Supplementary material for: An introductory biology research-rich laboratory course shows improvements in students’ research skills, confidence, and attitudes
Source: PLoS One. 2021 Dec 16;16(12):e0261278. doi: 10.1371/journal.pone.0261278 (PMC8675740; doi:10.1371/journal.pone.0261278)
Supplement: S4 File — (DOCX) [file pone.0261278.s004.docx]

BIO 112L **Daphnia Guided Lab Report**           Fall 2017

**GIVE A TITLE TO YOUR REPORT HERE THAT REPRESENTS YOUR QUESTION OR MAJOR FINDING (IF WELL SUPPORTED)**

- Think about what other researchers might find interesting or valuable in your work and what search terms they would use to find it

**Your name: Partner’s name: Group:**

- Type in your answers electronically and upload to Canvas.
- Use a *plain black font* (NOT **bold**, NOT *italics*, NOT with highlights, etc.).
- Formulate answers that are *concise*: specific, but brief and to the point.
- This write-up should be *your own work*. Any calculations, statistics, graphs, and write-ups should be your own.
- *Delete* the instructions in red *after* you are done and have made sure to address all points in red.

**I. Background information.**

**1. Experimental rationale.**

- Describe in 2-3 paragraphs making sure to address the points below in a coherent, readable write-up that shows the interest in the subject and the rationale.

#1. Put your work in context, describing the importance of the topic and the research.

- Show your interest in the subject, and why the reader should be interested.
- Demonstrate that you have researched the issue and learned new biology (see #2 below).

#2. Specific issues for Daphnia experiments.

- What is the plant and plant compound that you have chosen?
- How is it used and what is its importance for humans, including economically?
- Is there evidence that the compound 1) can affect the heart rate and/or 2) is toxic to insects or can serve as an insect deterrent and/or 3) is leached into the environment and may be an environmental toxin?
- Is there evidence for the mechanism by which it acts to affect the heart rate or physiology of the organism? Describe the physiology a bit.

#3. External sources

- Support your reasoning with information from external sources. Explain what is known, and what is not known, and why it would be important to know.
- Write in your own words. Best advice is to close the source and try to explain on your own, consulting with source as needed after first attempting on your own.
- Create in-text citations, in parenthesis at the end of each sentence, e.g. “Compound X has been found to increase heart rate in frogs (Smith, 1993)”. Make sure that your citations are also referenced at the end of this report (see instructions on reference list at the end).

**2.** **Experimental question and variables**.

a. What question was your experiment designed to answer? Be as specific as is appropriate for your case, for example, if the drug is known to increase/decrease heart rate in humans, or there is other indication of how it would affect heart rate, than say that. If there is not enough information either way, then it is not appropriate to be specific: just say that you are looking for an effect, be it decrease or increase.

b. What was the variable that you were aiming to understand the effect of? This is the *possible cause*, or *independent variable* (IV).

c. What were you *measuring*? This is the *effect*, or *dependent variable* (DV).

**II. Experimental procedure and results.**

**3. Plant compound formulation and preparation.**

- Describe the characteristics of the plant compound, including producer, specific amounts mentioned, other ingredients, whether it was a liquid or tablet.
- Describe how you **prepared the compound** for testing (any **dilutions**, in what, etc.). What is the final concentration (or of specific compounds, if those are specified on label).

**4. Experimental procedure**,

- Describe it clearly, step-by-step, so that anyone can reproduce your experiment.
- Describe and clearly identify any **control groups/set-ups (e.g., negative controls) or controlled variables** (for any possible or actual confounding factors that were controlled between groups).
- Describe clearly any **replicates** - repetitions of the same treatment.

**III. Experimental results.**

**5. Table.**

- The Table must clearly show how your experiment was conducted – **must match up** with both your Procedure & Graph.
- The table should show the calculation of the 1) **averages** and 2) **95% CI**.

**6. Graph.** Create a vertical bar graph in Excel as before (called “column graph” in Excel).

- Plot only **averages**, not measure for individual animals.
- Plot your baseline/control group **in the same graph** as the experimental treatment group.
- Each column should have its own **95%CI error bars** (going in both directions)
- Graph should overall follow the standards as described previously. Consider the scale/size of the graph to be such that the reader can **see clearly whether the error bars overlap or not**.
- The Graph must clearly show how your experiment was conducted – **must match up** with both your Experimental procedure & table.

**7. Statistical analysis.**

a. H_0_, Null hypothesis. State your null hypothesis. Remember, the null hypothesis is a statistical hypothesis stating there is no effect of the IV. It’s a *term* that is used in a very specific way.

b. Ha, alternative hypothesis. State your alternative hypothesis. Remember, the alternative hypothesis is a statistical hypothesis stating there is an effect of the IV.

c. Why is the paired t-test appropriate for this experiment? (Short sentence)

d. Report p value for two-tailed t-test.

**IV. Discussion.**

**8. Discussion of possible confounding factors.**

- Identify at least 8 possible confounding factors that could affect the DV in the experiment. Go through the checklist: you must address each of the categories in the numbered list; cross out an item when you have completed it.
- Confounding factors affect the DV (heart rate in this case), so be clear in your discussion in column 3 that it is the heart rate that you are concerned about.

1. Ingredients in your drug preparation other than the drug you are testing.
   - *If your compound was in pure spring water, just write exactly that in the 3^rd^ column and leave the 2^nd^ column blank.*
2. Do you expect any random or systematic fluctuations that might affect heart rate (for example, handling of the animals)?
   - *Explain your thoughts. You must be clear and specific as to what you mean here, not just “human factor”, what exactly do you think might have an effect?*
   - *No more than 3 of your reasons can be about this issue.*
3. Are all your experimental subjects identical? Are there any differences among them that may affect heart rate?
   - *No more than 3 of your reasons can be about this issue.*
4. Role of experimenter bias.
5. *Anything else? Remember, any variation between the control and experimental measurements may be a confounding variable for your experiment. Focus on ones that are most likely to affect heart rate.*

| **Possible confounding factor**  ***(explain, see above)*** | **Was CF a controlled variable? *Yes/No*.** | **How was confounding variable controlled? If not, how could it be controlled in future experiments?** |
| --- | --- | --- |
|  |  |  |
|  |  |  |
|  |  |  |
|  |  |  |
|  |  |  |
|  |  |  |

**9. Discussion of other limitations of the study.**

- Discuss 3 other issues relating to the execution of the experiment or otherwise that were limitations of the study. Focus on the ones that you have reason to believe are the most influential.

| **Study limitation** | **Describe the limitation in more detail and discuss why this is a problem.** |
| --- | --- |
| 1. Inaccurate heart rate measurements |  |
| 2. |  |
| 3. |  |

**10.** **Statistical analysis. Based on your statistical analysis, is there a statistically significant difference between your control and experimental treatment?**

a. Graph error bar analysis.

- Are the error bars overlapping or not? What does that mean? Explain clearly, showing an understanding of what error bars are.

b. t-Test analysis.

- First, answer the question, should the null hypothesis be accepted or rejected? Then, explain what that means as to whether there is a statistically significant difference between control and experiment. Use the p values from your t-test in your answer.
- Remember! In statistics, the null hypothesis is that there is no effect, that any differences are due to other factors, and not the IV in question. So, to **reject the null hypothesis** would mean that there **is** a statistically significant difference!

**11. Overall conclusions from experiment & future direction.**

- First, use points 8-11 above to **summarize in 1-2 paragraphs** what are the possible reasons why you may have not been able to determine an effect when there was one– or determined an effect when there was none. Summarize the factors that affected your overall conclusion. Don’t be mechanical here, but aim to create a coherent, readable write-up that meaningfully reports on your experiment.
- Second, **formulate a very careful conclusion**, based on the totality of all considerations. What were the *biggest factors* that limited your ability to formulate a reliable conclusion? Remember, there were significant limitations to your study! Also remember that statistical analysis is only as good as the experiment – it cannot make a bad experiment good! Showing a statistically significant difference when the experiment has major flaws means you do not know whether there was an actual difference.
- Third, discuss what could be done in **future experiments** to overcome the biggest challenges in this experiment. Identify at least 2 such factors.

**12. Daphnia as a model organism.**

a. What are 2 advantages of using Daphnia as a research subject?

b. Daphnia as model organism to study the effects of drugs on human physiology. Describe at least 1 advantage and at least 1 disadvantage in this respect.

c. Daphnia as model organism to study the environmental effects of drugs. Describe at least 1 advantage and at least 1 disadvantage in this respect.

**13. Reference list.**

- Provide here the full reference for any in-text citations (see also “I.1.Experimental rationale”)
- Use the citation format described in <http://uncg.libguides.com/c.php?g=83192&p=2634289>.
  - Note that a scholarly article found online and an internet source are cited differently.
  - Under each reference, note in **bold** whether the article is primary or secondary/review.
- You need to to have at least 5 scholarly references (primary or secondary/review, with at least 2 primary articles).

1.

2.

3.

4.

5.

BIO 112L **Caterpillar Guided Lab Report**           Spring 2018

| **Your name:** |  |
| --- | --- |
| **Name(s) of team member(s):** |  |

- Type in your answers electronically and upload to Canvas.
- Use a *plain black font* (NOT **bold**, NOT *italics*, NOT with highlights, etc.).
- Don’t alter the headings in bold – they help us find and give credit for each component of the report.
- Your answers should be *concise*: specific, but brief and to the point.
- Refer back to the handout on group vs. individual work. **This write-up should be *your own work*. Any calculations, statistics, graphs, and write-ups should be your own.** Ask instructor for help.
- Don’t be mechanical here to “get it done”; aim instead to create a coherent, readable write-up that meaningfully reports on your experiment. Make sure to give extra attention - careful thought and editing time - for the Discussions section (especially conclusions under #12).
- *Delete* all instructions in red *after* you are done with that section and **have made sure to address all points in red. Consult the Canvas grading Lab Report rubrics to help you meet the standards for this lab report.**

**I. Introduction**

**1. Experimental question and variables for the caterpillar feeding and growth experiment**.

What variable(s) were you trying to determine the effect of? These are the *possible causes*, or *independent variable* (IV).

| IV (independent variable) |  |
| --- | --- |

What were you *measuring*? This is the *effect*, or *dependent variable* (DV).

| DV (dependent variable) |  |
| --- | --- |

State your null hypothesis. Make sure to link IV to DV. Remember, the “null hypothesis” is a statistical hypothesis stating there is no effect of the IV on the DV. It’s a *term* that is used in a very specific way.

| H_0_ (null hypothesis) |  |
| --- | --- |

State your alternative hypothesis. Make sure to link IV to DV. Remember, the “alternative hypothesis” is a statistical hypothesis stating there is an effect of the IV on the DV.

| H_a_ (alternative hypothesis) |  |
| --- | --- |

**2. Experimental rationale for the caterpillar feeding and growth experiment.**

Describe in a couple of paragraphs things you considered in developing your feeding and growth experimental design, including:

- what is known about the caterpillar life cycle and food eaten by caterpillars in the wild.
- how chemical plant defenses can affect caterpillar feeding and growth.
- your reasons for choosing the particular plant-derived compound you studied and what is known and unknown about the compound and its effects on caterpillars and other animals.

For full credit, you must demonstrate that you have learned about caterpillars and plant defenses, and have thoughtfully applied it to your experimental design in what you did (and didn’t do). Use the Chemical Warfare reading and sources from your library research. Cite them in the text.

Using external sources:

- Support your reasoning with information from external sources that are scholarly, peer-reviewed, articles. Explain what is known, and what is not known, and why it would be important to know.
- Write in your own words. Best advice is to close the source and try to explain on your own, consulting with source as needed after first attempting on your own.
- Create in-text citations, in parenthesis at the end of each sentence, e.g. “Compound X has been found to increase heart rate in frogs (Smith, 1993)”.  Make sure that your citations are also referenced at the end of this report.

**II. Experimental procedure.**

**3. Plant compound formulation and preparation.**

Describe the name and form (solvent, concentration) of your compound. Describe how you prepared the compound for testing (any dilutions, in what, if you prepared an extract, how did you do it, etc.).

| **Compound name** | **Solvent** | **Concentration** | **Preparation** |
| --- | --- | --- | --- |
|  |  |  |  |

**4. Control and experimental groups, and the controlled variables for the feeding and growth experiment.**

In a perfectly controlled experiment, the only difference between the control and experimental groups would be the IV. All other possible confounding variables will be kept the same between the two groups. Identify at least 6 possible confounding variables between the two groups that you attempted to control – these are your controlled variables- and discuss for each whether you were able to have them be similar between the two groups.

| **Experimental group** | **Control group** |
| --- | --- |
|  |  |
| **Controlled Variable** | **Was the variable successfully controlled?**  **If not, what was the problem or difference?** |
| **1.** |  |
| **2.** |  |
| **3.** |  |
| **4.** |  |
| **5.** |  |
| **6.** |  |

**5. Experimental procedure for the caterpillar feeding and growth experiment.**

Insert your Methods; be sure to make any improvements and clarifications based on feedback from the previous assignment. Describe your experimental procedure in **paragraph form**. Make sure to write **clearly**, while being very **specific** as to all important details, so that anyone can reproduce your experiment. Please write in the **third person, past tense**, as this is the standard for writing experimental procedures. Describe clearly any **replicates** - repetitions of the same treatment (be it control or experimental). Be clear whether your replicates used the same caterpillars, or new ones for each replicate.

**III. Results**

**6. Table of results for the feeding and growth experiment.**

Insert the table with your recorded observations and measurements. Make any improvements and corrections as needed from the table you recorded previously in Excel. Make sure your table is sized and formatted appropriately after being inserted into this document. Check all of the following:

- Above each table should be an informative **title,** immediately followed by a short **description** of 1-3 sentences that summarize it (e.g., what was done, including any replicates or statistics). For example, “**Table 1. Effect of different disinfectants on density of bacteria present on tables in kindergarten classrooms.** Clorox was 2X more effective than other disinfectants (p<0.05). Density was sampled 30 minutes after use on 15 tables.”
- The Table must clearly show how your experiment was conducted – **must match up** with both your Procedure & Graph.
- The table should show the calculation of the **averages** from replicates.
- The table should also include the 95% CI.

Annotate the table with any notes (below) that clarify the data collected and any notable observations that are not included in table (or create a separate column in your table for notes on your observations).Insert the table with your recorded observations and measurements.

**7.** **Graph of feeding and growth experiment.**

Insert the vertical bar graph that you created in Excel (called “column graph” in Excel). Make any improvements and corrections from your previous assignment. Make sure your graph is sized and formatted appropriately after being inserted into this document. Be sure to follow the overall standards described in the first graphing assignment and in class. Check all of the following:

- Below each graph should be an informative **title,** immediately followed by a short **description** of 1-3 sentences that summarize it (e.g., what was done, including any replicates or statistics). For example, “**Figure 1. Effect of different disinfectants on density of bacteria present on tables in kindergarten classrooms.** Clorox was 2X more effective than other disinfectants (p<0.05). Density was sampled 30 minutes after use on 15 tables.”
- Always plot **averages** on the graph.
- The graph must include 95% CI error bars for both the control and experimental groups.
- The Graph must clearly show how your experiment was conducted – **must match up** with both your Experimental procedure & table.

**8. Statistical analysis for feeding and growth.** Record the results of the t-test here.

|  | p-value | Is the t-test statistic smaller than the critical value p=0.05? (yes or no) |
| --- | --- | --- |
| Feeding and growth |  |  |

**Describe the t-test used (1 vs 2 tailed, paired vs unpaired) and explain why this is the appropriate test.**

**Based on the t-test values alone, should you reject H_0_ (Yes/No)?**

**If yes, what was the effect of the compound?**

**IV. Discussion.**

**9. Caterpillars as a model organism.**

We used caterpillars as a model organism to study the effects of a plant-derived compound on feeding and growth. List and discuss at least 2 advantages and at least 2 disadvantages in using caterpillars as the model organisms in this study. When you’re thinking of advantages or disadvantages, compare them to other organisms.

| **Caterpillars as the model organism in feeding/growth experiments** | **Description** |
| --- | --- |
| **Advantages** | **1.** |
|  | **2.** |
| **Disadvantages** | **1.** |
|  | 2. |

**10. Discussion of experimental design issues affecting your ability to make accurate observations of differences in feeding and/or growth.**

List at least three confounding factors or additional reasons that impact your ability to make accurate observations. Describe **how** each could affect your ability to make accurate observations or reach conclusions of differences in feeding and/or growth.

| **Possible issues affecting accuracy of observations of feeding/growth.** | **Explain how the issue compromised your ability to reach accurate conclusions.** |
| --- | --- |
|  |  |
|  |  |
|  |  |

**11. Discussion of statistical analysis of feeding and growth experiment.**

**a. Graph error bar analysis.**

Are the error bars overlapping or not? What does that mean? Explain clearly, showing an understanding of what error bars are.

**b. t-test analysis.**

First, answer the question, should the null hypothesis be accepted or rejected? Then, explain what that means as to whether there is a statistically significant difference between control and experiment. Use the p values from your t-test in your answer. Remember! In statistics, the null hypothesis is that there is no effect, that any differences are due to other factors, and not the IV in question. So, to **reject the null hypothesis** would mean that there **is** a statistically significant difference!

**c. Overall conclusion.** Based on your statistical analyses, is there a statistically significant difference between your control and experimental treatment?

**12. Conclusions: Feeding and Growth Experiment.**

**This section is very important. It reflects the conclusion as warranted by the data gathered – which can support one hypothesis over another, or can be inconclusive. Demonstrate comprehensively your reasoning by linking specific data or analyses to specific conclusions. Make sure to reflect on and edit this section before submitting.**

Based on **all** of the considerations, did the compound have an effect on feeding and/or growth? First, formulate a very careful conclusion that is based on the totality of all considerations that you considered in points 1-4. Is the evidence strongly or weakly supportive of the alternative hypothesis or is there no discernable effect? Explain how you formed your conclusion.

What aspects of how the experiment was conducted makes it difficult to make a definitive conclusion? Remember, statistical analysis is only as good as the experiment – it cannot make a bad experiment good! If there were major flaws in the experiment that hinder interpretation, you must state that your results are inconclusive and explain why.

**13. Future directions.**

Discuss what could be done in future experiments to overcome the biggest challenges in this experiment. Identify at least 2 such factors. Don’t worry about repeating yourself because you have already said those things in 1-6; purposeful repetition is *required* for you to demonstrate your careful, evidence-based, thinking.

**V. Reference list.**

Provide the full reference for any in-text citations. Use the citation format described in <http://uncg.libguides.com/c.php?g=83192&p=2634289>. You **must** use **at least 3 scholarly, peer-reviewed** (primary or secondary, review) articles (additional popular science website references may be appropriate for generally known facts such as caterpillar life-cycle). Note that a scholarly article found online and an internet source are cited differently. Under each reference, note in **bold** whether the article is primary or secondary/review.
