# Supplementary material for: An introductory biology research-rich laboratory course shows improvements in students’ research skills, confidence, and attitudes
Source: PLoS One. 2021 Dec 16;16(12):e0261278. doi: 10.1371/journal.pone.0261278 (PMC8675740; doi:10.1371/journal.pone.0261278)
Supplement: S5 File — (DOCX) [file pone.0261278.s005.docx]

Lab report (with key)

Q1Last name, First name

Q3What was the Independent Variable (IV) in the Daphnia experiment? (word or phrase)

**0.5p**

**Drug/compounds of choice (may mention their particular experiment or say caffeine/ethanol)**

Q4 What was the Dependent Variable (DV) in the Daphnia experiment? (word or phrase)

**0.5p**

**Heart rate**

Q5 What were 2 possible Confounding Variables in the Daphnia experiment? (a couple of words or phrases)

**1p**

**Reasonable possible confounding variables in the experiment that could or did impact heart rate**

**Description of how it is a confounding variable impacting results is not required**

Q6 The outcome that one measures in an experiment is the...

**1p**

- - Independent Variable (IV)
  - **Dependent Variable (DV)**
  - Confounding variable

Q7 Which is the dependent variable (DV) in the Biodiversity/Peabody Park (first) lab experiment?

**1p**

- - light level
  - **number of soil arthropods**
  - soil composition
  - type of habitat

Q8 Which is the dependent variable (DV) in the Caterpillar lab experiment?

**1p**

- - caterpillar genetic differences
  - **caterpillar mass**
  - compound tested
  - temperature

Q9 Which variable is plotted on the Y axis?

**1p**

- - Independent Variable (IV)
  - **Dependent Variable (DV)**
  - Confounding variable

Q10 The reason we are concerned with confounding variables is because they impact the...

**1p**

- - Independent Variable (IV)
  - **Dependent Variable (DV)**

Q11 In a controlled experiment, the experimenter deliberately changes which variable between the control group and the experimental group?

**1p**

- - **Independent Variable (IV)**
  - Dependent Variable (DV)
  - Confounding variable

Q14 Certain organisms – “model organisms” – are chosen to conduct experiments on because of the advantages they provide. However, they are not perfect and may have disadvantages as well, or may be better suited for some experiments over others. Demonstrate that this is the case, by using examples of model organisms from class. Make sure you mention at least 1 advantage and 1 disadvantage. 1-2 of sentences are sufficient here.

**3p**, **1.5p for each of 2 considerations. Anything that is true about the model organisms. Can use the same organism for both advantage and disadvantage.**

Q13 An experiment is performed to test the effect of Supplement X on weight loss. It is found that the participants that took Supplement X lost on average 2.5 pounds over two weeks. Why can't you make a conclusion based on this information that Supplement X helps with weight

loss? Focus on your top consideration/s. 1-2 of sentences are sufficient here.

**2p**

**1p – identify confounds**

**1p – explanation**

Q15 An experiment was conducted, and the experimenter found that the averages in the control versus the experimental groups are different. Why is it difficult to reach a correct conclusion by comparing only averages? *1-2 of sentences are sufficient here.*

**Averages do not convey information about distributions of values in sample collected or where the population mean is (relative to sample mean). Accept either formulation (relating to SD or to SE).**

**2p**

**1p – idea**

**1p – explanation**

Q16 An experiment was conducted, and a standard deviation of 136.4 was calculated. Show your reasoning of how you will judge whether this is a large SD or not. Focus on your top consideration/s. 1-2 of sentences are sufficient here.

**There is not enough information – would have to know what average is. If SD is small relative to average, there’s very little variation.**

**2p**

**1p – idea**

**1p – explanation**

Q17 The results of an experiment are presented below, with averages plotted in the bar graphs, and error bars representing standard error calculations for each group. What is the best conclusion that can be drawn from this experiment? *Choose the best answer.*

**1p**


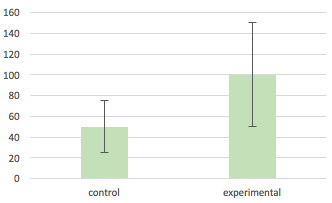


- - The IV affects the control group, but not the experimental group
  - The IV affects the experimental group, but not the control group
  - There is a statistically significant difference between the two groups
  - There is **no** statistically significant difference between the two groups

Q18. An experiment was performed with a control and an experimental group, and the p-value for the t-test was calculated as being 0.1. What is the best conclusion that can be drawn from this experiment, at the 0.05 confidence level? *Choose the best answer.*

**0.5p**

- - There is a statistically significant difference between the two groups
  - There is **no** statistically significant difference between the two groups

Q19 An experiment was performed with a control and an experimental group, and the p-value for the t-test was calculated as being 0.1. What is the best conclusion that can be drawn from this experiment, at the 0.05 confidence level? The scenario and numbers are the same as in the previous question. *Choose the best answer.*

**0.5p**

- There is a statistically significant difference between the two groups because there is a 10% chance the results are due to chance/other factors
- There is a statistically significant difference between the two groups because there is a 90% chance the results are due to chance/other factors
- There is **no** statistically significant difference between the two groups because there is a 10% chance the results are due to chance/other factors
- There is **no** statistically significant difference between the two groups because there is a 90% chance the results are due to chance/other factors

**19p total**
