# Supplementary material for: An introductory biology research-rich laboratory course shows improvements in students’ research skills, confidence, and attitudes
Source: PLoS One. 2021 Dec 16;16(12):e0261278. doi: 10.1371/journal.pone.0261278 (PMC8675740; doi:10.1371/journal.pone.0261278)
Supplement: S6 File — (DOCX) [file pone.0261278.s006.docx]

Lab Report Coding Rubric

| **Category** | **Comments** |
| --- | --- |
| 1. Clearly states research question or hypothesis | Students are prompted in template |
| 1. Correctly identifies independent variable | Students are prompted in template  No points if they just repeat 1. |
| 1. Correctly identifies dependent variable | Students are prompted in template |
| 1. Indicates how dependent variable is measured (for example, HR/Mass or length) | Students are prompted in template  Daphnia: Heart Rate, Caterpillar: mass or length not just growth |
| 1. Distinguishes between control and experimental groups | Daphnia: Procedure. Students must clearly identify differences between baseline/control and experimental/treatment – but do not need to say “control” or link explicitly.  Caterpillar: Table that prompts. Look in Methods, Graph if not. |
| 1. Identifies at least one relevant confounding factor | CF as variable affecting the outcome/DV. No need to explain why it is a confounding factor. Anything that could be a possible CF. |
| 1. Identifies controlled variables | Describe conditions that are controlled between the two groups or baseline and treatment conditions.  Daphnia: Table that prompts.  Caterpillar: Table that prompts more directly than Daphnia. |
| 1. X & Y axes are plotted and labeled correctly | For a score, need both X and Y axes |
| 1. X & Y have correct units (where appropriate) | Bar graph – units for DV on Y axis. |
| 1. Error bars are present |  |
| 1. Correctly uses p value to accept/reject null hypothesis | In Results, have to explain some. If not, has to be clear from conclusion/discussion that they understand the framework. |
| 1. Clearly states conclusion | Okay if discuss complexity of data vs. error bars vs. statistics (which could be contradictory to each other in some students’ experiments) |
| 1. Uses statistical analysis to qualify conclusions | Student must be linking statistical analysis to qualify conclusion in their answer. |
| 1. Correctly explains how statistical analysis supports or refutes hypothesis | Explain correctly link with results. In Conclusions. If not, in Statistical Results, look for linking statistics to conclusion. |
| 1. Recognizes the impact of confounding factors in qualifying conclusions | Has to discuss the effect in making conclusions of at least one actual CF, and not a problem with experiment. |
| 1. Recognizes the limitations of the experiment when drawing conclusions | Any reasonable limitations. |
| **TOTAL SCORE: 16** |  |

**Comments:**
