# Supplementary material for: An introductory biology research-rich laboratory course shows improvements in students’ research skills, confidence, and attitudes
Source: PLoS One. 2021 Dec 16;16(12):e0261278. doi: 10.1371/journal.pone.0261278 (PMC8675740; doi:10.1371/journal.pone.0261278)

Lab Exam Coding Rubric

|  | Question/prompt | Answer | Format | Points |
| --- | --- | --- | --- | --- |
| Q3 | 1. What was the Independent Variable (IV) in the Daphnia (F17)/Caterpillar (S18) experiment? (word or phrase) | Drug/compounds of choice | W^1^ | 1 |
| Q4 | 2. What was the Dependent Variable (DV) in the Daphnia (F17)/Caterpillar (S18) experiment? (word or phrase) | Heart rate (F17) Growth/development/mass/food preference (S18) | W | 1 |
| *Q5* | *What were 2 possible Confounding Variables in the Daphnia experiment? (a couple of words or phrases)* | *Not scored. Students were not prompted to explain and it was hard to tell whether they are correctly identifying variables that were confounding to the DV.* | *W* |  |
| Q6 | 3. The outcome that one measures in an experiment is the... | Dependent Variable (DV) | MC^2^ | 1 |
| Q7 | 4. Which is the dependent variable (DV) in the Biodiversity/Peabody Park (first) lab experiment? | number/type of soil arthropods | MC | 1 |
| Q8 | 5. Which is the dependent variable (DV) in the Caterpillar lab experiment? | caterpillar mass | MC | 1 |
| Q9 | 6. Which variable is plotted on the Y axis? | Dependent Variable (DV) | MC | 1 |
| Q10 | 7. The reason we are concerned with confounding variables is because they impact the… | Dependent Variable (DV) | MC | 1 |
| Q11 | 8. In a controlled experiment, the experimenter deliberately changes which variable between the control group and the experimental group? | Independent Variable (IV) | MC | 1 |
| Q14 | 9. Certain organisms – “model organisms” – are chosen to conduct experiments on because of the advantages they provide. However, they are not perfect and may have disadvantages as well, or may be better suited for some experiments over others. Demonstrate that this is the case, by using examples of model organisms from class. Make sure you mention at least 1 advantage and 1 disadvantage. 1-2 of sentences are sufficient here. | 1p for each of 2 considerations | W | 2 |
| Q13 | 10. An experiment is performed to test the effect of Supplement X on weight loss. It is found that the participants that took Supplement X lost on average 2.5 pounds over two weeks. Why can't you make a conclusion based on this information that Supplement X helps with weight loss? Focus on your top consideration/s. 1-2 of sentences are sufficient here. | 1p – identify confounds  1p – explanation | W | 2 |
| Q15 | 11. An experiment was conducted, and the experimenter found that the averages in the control versus the experimental groups are different. Why is it difficult to reach a correct conclusion by comparing only averages? *1-2 of sentences are sufficient here.* | Answers describe either that averages do not convey information about distributions of values in the sample collected (e.g., outliers, SD) or where the population mean is relative to sample mean (e.g., SE).  1p – idea  1p – explanation | W | 2 |
| Q16 | 12. An experiment was conducted, and a standard deviation of 136.4 was calculated. Show your reasoning of how you will judge whether this is a large SD or not. Focus on your top consideration/s. 1-2 of sentences are sufficient here. | There is not enough information – would have to know what average is. If SD is small relative to average, there’s very little variation.  1p – idea  1p – explanation (show how this SD could be small or large depending on the values). | W | 2 |
| Q17 | 13. The results of an experiment are presented below^3^, with averages plotted in the bar graphs, and error bars representing standard error calculations for each group. What is the best conclusion that can be drawn from this experiment? *Choose the best answer.* | There is no statistically significant difference between the two groups | MC | 1 |
| Q18. | 14. An experiment was performed with a control and an experimental group, and the p-value for the t-test was calculated as being 0.1. What is the best conclusion that can be drawn from this experiment, at the 0.05 confidence level? *Choose the best answer.* | There is no statistically significant difference between the two groups | MC | 1 |
| Q19 | 15. An experiment was performed with a control and an experimental group, and the p-value for the t-test was calculated as being 0.1. What is the best conclusion that can be drawn from this experiment, at the 0.05 confidence level? The scenario and numbers are the same as in the previous question. *Choose the best answer.* | There is no statistically significant difference between the two groups because there is a 10% chance the results are due to chance/other factors | MC | 1 |
|  |  |  | Total  W  MC | 19  10  9 |

^1^W – written answers

^2^MC – multiple-choice format

^3^Graph:


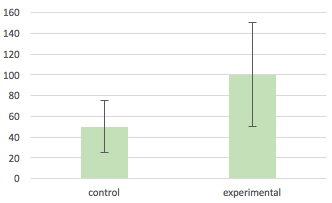

Supplement: S7 File — (DOCX) [file pone.0261278.s007.docx]
