# Supplementary material for: An introductory biology research-rich laboratory course shows improvements in students’ research skills, confidence, and attitudes
Source: PLoS One. 2021 Dec 16;16(12):e0261278. doi: 10.1371/journal.pone.0261278 (PMC8675740; doi:10.1371/journal.pone.0261278)
Supplement: S8 File — (DOCX) [file pone.0261278.s008.docx]

**E-EDAT coding rubric**

E-EDAT scored according to Brownell et al., 2013. Comments below are as it applies to the context of the study.

Difference is in item 8b in Brownell et al, 2013 (wording related to “proving a hypothesis”) which we replaced with 8.3 and 8.4.

| **Category** | **Max pts** | **Abbreviation** | **Comments** |
| --- | --- | --- | --- |
| 1. Identifies variable which will be manipulated | 1 | IV | Must be clear what experiment is about, thought does not have to name as IV or DV. |
| 1. Identifies variable which will be measured | 1 | DV |  |
| 1. Describes how dependent variable will be measured | 2 | measure | 1p. Describe how endurance will be evaluated, but vague  2p. More specific - per unit of time or distance, or implies "to exhaustion," any range, level, amount or concentration for physiological measures. |
| 1. Realization that other variables need to be held constant | 2 | Controlled var. | 1p. Mentions one variable. See detailed list in published rubric.  2p. >2 variables described, indicating systematic consideration.  Accept if limit to certain group (age, gender, athletes). |
| 1. Placebo or vehicle effect (e.g., control group similar) | 2 | Placebo, Control, or Vehicle effect | 1p. Mentions control or placebo group.  1p. Above + Explain correctly placebo effect OR describe as double-blinded OR very clearly identify placebo group/identify vehicle effect (water vs. water + ginseng) |
| 1. Sample size | 2 | Large sample size | 1p. States “large” or gives a specific number >10.  1p. Above + Explanation. Specific reason that is correct. |
| 7a. Repeat experiments | 1 | Repeat exp. | 1p. States need to repeat experiment. Do not accept if only propose a longer-term study to collect more participants. |
| 7b. Reasoning for repeating experiment | 2 | Repeat – expl. | 1p. Explains, but vaguely  1p. Above + Clear, correct explanation. |
| 8.1. Conclusion - stated | 1 | Concl. -stated | 1p. State conclusion. Get 1p even if states conclusion with certainty. (See 8.2 below). “If X, then Y” |
| 8.2. Conclusion – showing uncertainty in conclusion. | 1 | Concl - uncertainty | 1p. Indicates uncertainty in conclusion. Get 1p if uses qualifiers “may, could be, supports” or if states certainty increases under certain conditions, even if it is vague (See 8.3 below). Do not accept if just that uncertain about whether get +/- results. |
| 8.3. Conclusion – explanation for uncertainty in conclusion | 1 | Concl. - explanation | 1p. Gives one or more reasons that explain why one cannot have certainty in conclusion. This could be a discussion of confounds, practical issues or other limitations. Answer must explicitly link considerations to conclusion. |
| 8.4. Conclusion - statistics | 1 | Concl- stat | 1p. Mentions that statistical analysis will be used to make conclusions. |
| **TOTAL SCORE:** | 17 |  |  |

**Comments:**
