# Supplementary material for: An introductory biology research-rich laboratory course shows improvements in students’ research skills, confidence, and attitudes
Source: PLoS One. 2021 Dec 16;16(12):e0261278. doi: 10.1371/journal.pone.0261278 (PMC8675740; doi:10.1371/journal.pone.0261278)
Supplement: S10 File — (DOCX) [file pone.0261278.s010.docx]

**S10 Data file. Statistical analyses.**

**SRBCI**

**SRBCI Summary**

| Cohort | N | Control *M* (*sd*) | Intervention *M* (*sd*) | t-test | Effect size: *d* | Wilcoxon |
| --- | --- | --- | --- | --- | --- | --- |
| Fall All^1^ | 207 | .34 (1.95) | 1.70 (2.32) | 3.96*** | .64 | -4.03*** |
| Fall Matched^2^ | 86 | .19 (2.04) | 1.65 (2.32) | 3.11** | .67 | -3.27** |
| Spring All^3^ | 218 | .18 (1.99) | 1.39 (2.36) | 3.59*** | .55 | -2.68** |
| Spring Matched^4^ | 80 | .15 (2.21) | 1.33 (2.40) | 2.28* | .51 |  |
| Combined All^5^ | 426 | .26 (1.97) | 1.54 (2.33) | 4.84*** | .60 | -4.71*** |
| Combined Matched^6^ | 166 | .17 (2.11) | 1.49 (2.35) | 3.82*** | .59 | -4.10*** |
| **p* < .05, ***p* < .01, *** *p* < .001  ^1-3^Control violated normality  ^4^Met all assumptions  ^5^Violated normality & homogeneity of variances  ^6^Violated normality | | | | | | |

**SRBCI Pre-Post Means and Standard Deviations**

|  |  | Control | | Intervention | |
| --- | --- | --- | --- | --- | --- |
| Cohort | N | Pre *Mean* (*sd*) | Post *Mean* (*sd*) | Pre *Mean* (*sd*) | Post *Mean* (*sd*) |
| Fall All^1^ | 207 | 3.90 (1.72) | 4.24 (1.93) | 4.05 (1.72) | 5.75 (2.75) |
| Fall Matched^2^ | 86 | 4.21 (1.64) | 4.40 (2.03) | 4.07 (1.74) | 5.72 (2.77) |
| Spring All^3^ | 218 | 4.03 (1.85) | 4.21 (1.86) | 3.94 (1.77) | 5.33 (2.21) |
| Spring Matched^4^ | 80 | 4.40 (2.15) | 4.55 (2.32) | 4.03 (1.89) | 5.35 (2.27) |
| Combined All^5^ | 426 | 3.97 (1.79) | 4.22 (1.89) | 3.99 (1.74) | 5.52 (2.47) |
| Combined Matched^6^ | 166 | 4.30 (1.89) | 4.47 (2.16) | 4.05 (1.80) | 5.54 (2.53) |

**SRBCI Covariates**

| Cohort | Sex  (*F*, η^2^) | Race  (*F*, η^2^) | Sex*Race  (*F*, η^2^) |  |
| --- | --- | --- | --- | --- |
| Fall All | .82 (.004) | .53 (.005) | 2.88 (.028) | Failed normality for all conditions |
| Fall Matched | .15 (.002) | 1.34 (.032) | .64 (.016) | Failed normality for all conditions & variances |
| Spring All | 2.48 (.012) | .09 (.001) | .76 (.007) | Failed normality (except Black) |
| Spring Matched | .98 (.013) | .60 (.016) | .25 (.007) | Met normality (except Other race) |
| Combined All | .62 (.001) | .18 (.001) | 2.38 (.011) | Failed normality for all conditions |
| Combined Matched | .06 (.011) | .21 (.003) | .75 (.009) | Failed normality (except White) |

**p* < .05, ***p* < .01, *** *p* < .001

-Used Shapiro-Wilk for normality and Levene’s test for homogeneity of variances

**E-EDAT**

**Learning gains - Fall 2017**

For the control group, there were a total of 13 students who had a positive Pre-Post difference and a total of 24 students who had a negative Pre-Post difference (this might just be noise since most negative differences are small).


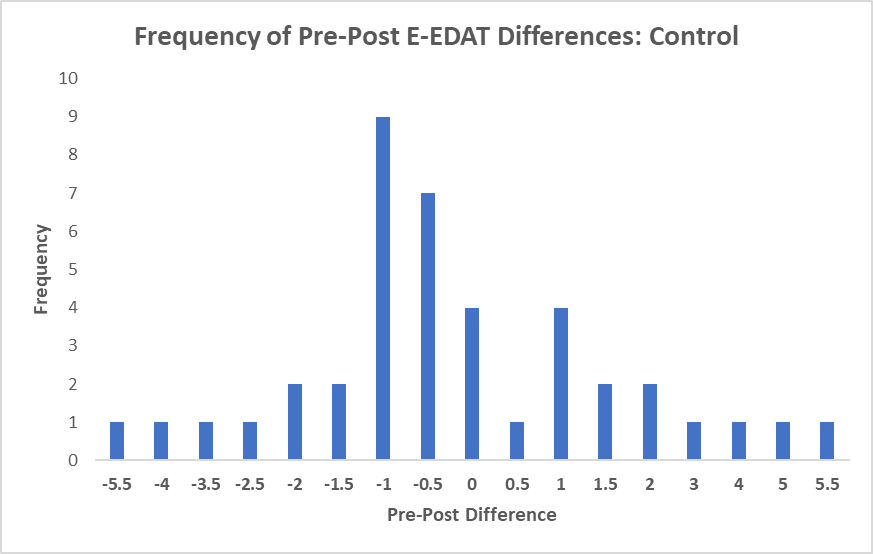


For the intervention group, there were a total of 31 students who had a positive Pre-Post difference and a total of 11 students who had a negative Pre-Post difference.


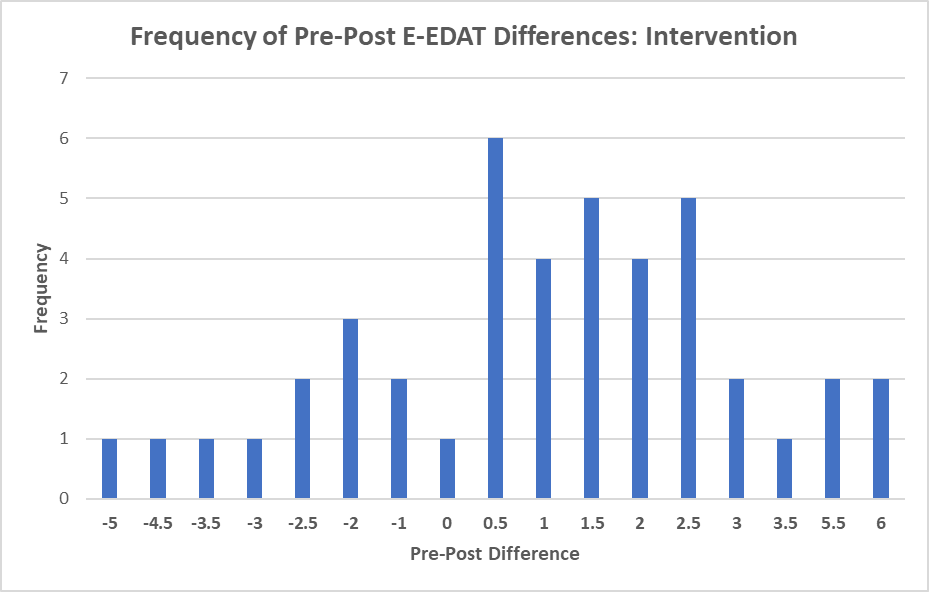


**Learning gains - Spring 2018**

For the control group, there were a total of 15 students who had a positive Pre-Post difference and a total of 23 students who had a negative Pre-Post difference (this might just be noise since most negative differences are small).


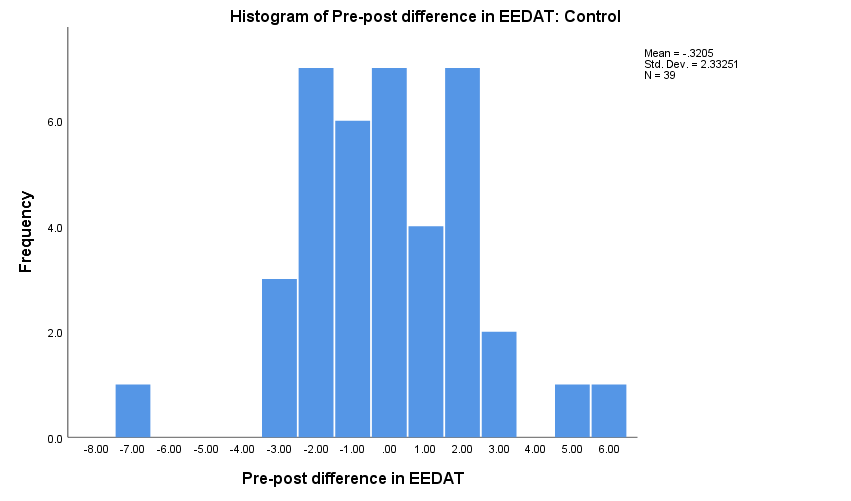


For the intervention group, there were a total of 20 students who had a positive Pre-Post difference and a total of 16 students who had a negative Pre-Post difference.


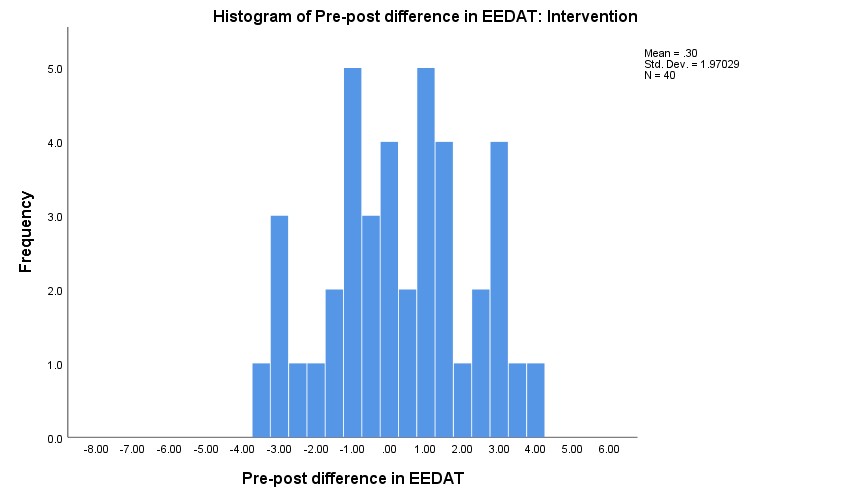


**Learning gains – Combined**

For the control group, there were a total of 28 students who had a positive Pre-Post difference and a total of 47 students who had a negative Pre-Post difference (this might just be noise since most negative differences are small).


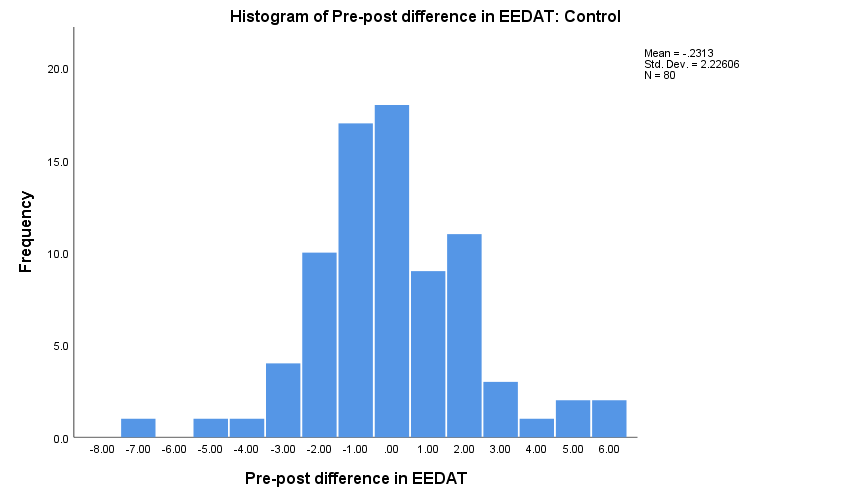


For the intervention group, there were a total of 51 students who had a positive Pre-Post difference and a total of 27 students who had a negative Pre-Post difference.


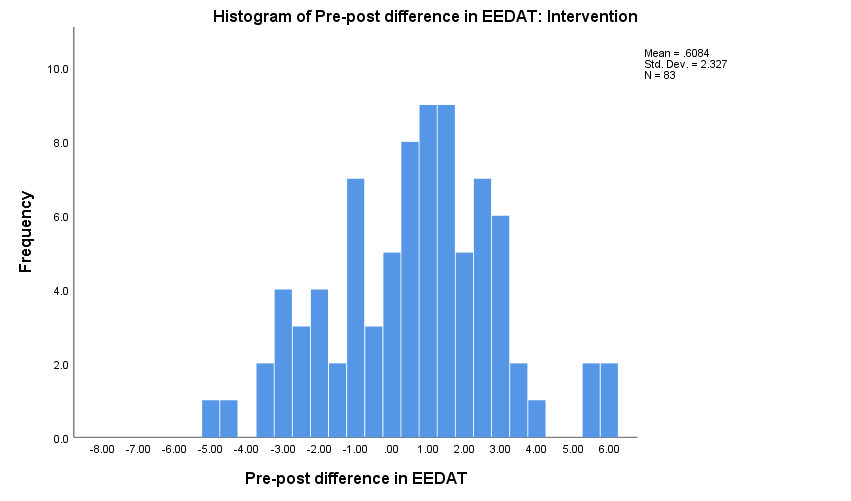


Repeated measures ANOVA to examine main effect of time and/or group, as well as interaction between time and group

| **Cohort** | **Control Difference (Post – Pre)** | **Intervention Difference (Post - Pre)** | **Time Effect**  **(*F*)** | **Group Effect**  **(*F*)** | **Interaction (Time * Group)**  **(*F*)** |
| --- | --- | --- | --- | --- | --- |
| Fall | -0.15 | +0.90 | 2.06 | 6.75* | 3.98* |
| Spring | -0.32 | +0.30 | .002 | 4.94* | 1.64 |
| Combined | -0.23 | +0.61 | 1.12 | 11.63** | 5.54* |
| **Significant at the *p* < .01 level (2-tailed)  *Significant at the *p* < .05 level (2-tailed) | | | | | |

Examine if intervention (+.61) had a significant pre-post difference from 0 (instead of -.23 points from control group)

| **Group Statistics** | | | | | |
| --- | --- | --- | --- | --- | --- |
|  | GROUP | N | Mean | Std. Deviation | Std. Error Mean |
| SCORE_COMP | Control | 83 | .0000 | 2.32700 | .25542 |
|  | Intervention | 83 | .6084 | 2.32700 | .25542 |

| **Independent Samples Test** | | | | | | | | |
| --- | --- | --- | --- | --- | --- | --- | --- | --- |
|  | | t-test for Equality of Means | | | | | | |
|  |  | t | df | Sig. (2-tailed) | Mean Difference | Std. Error Difference | 95% Confidence Interval of the Difference | |
|  |  |  |  |  |  |  | Lower | Upper |
| SCORE_COMP |  | -2.382 | 164 | .018 | -.60843 | .25542 | -1.11277 | -.10410 |

The intervention group had an EEDAT mean difference significantly greater from 0, indicating some evidence of pre-post learning *t*(164) = 2.38, *p* =.018, *d* = .26. With Cohen’s *d* = .26, there was a small to moderate effect of learning.

**CLASS-Bio**

**Overall Percentage Change: Favorable Responses**

| Cohort | N | Control *M* (*sd*) | Intervention *M* (*sd*) | t-test | Effect size: *d* | Wilcoxon | MANOVA | Effect size: η^2^ |
| --- | --- | --- | --- | --- | --- | --- | --- | --- |
| Fall All^1^ | 208 | -.41 (15.19) | .30 (12.09) | .29 | .05 | -.35 | .87 | .03 |
| Fall Matched^2^ | 86 | .93 (14.66) | .23 (12.22) | -.24 | -.05 | -.30 | .74 | .06 |
| Spring All^3^ | 218 | -4.99 (16.35) | 2.59 (16.92) | 2.83** | .46 | -1.98* | 1.28 | .04 |
| Spring Matched^4^ | 80 | -5.67 (16.66) | 4.58 (14.96) | 2.90** | .65 | -.55 | 1.66 | .139 |
| Combined All^5^ | 426 | -2.73 (15.93) | 1.51 (14.80) | 2.30* | .28 | -1.21 | 1.30 | .02 |
| Combined Matched^6^ | 166 | -2.25 (15.91) | 2.33 (13.70) | 1.99* | .31 | -.43 | 1.20 | .05 |
| **p* < .05, ***p* < .01  ^1,3-6^Failed normality  ^2^Control Failed normality | | | | | | | | |

**Overall Percentage Change of Favorable Responses: Covariates**

| Cohort | Sex  (*F*, η^2^) | Race  (*F*, η^2^) | Sex*Race  (*F*, η^2^) |  |
| --- | --- | --- | --- | --- |
| Fall All | 1.01 (.005) | .01 (.000) | 1.24 (.012) | Failed normality (except male) |
| Fall Matched | .93 (.011) | .04 (.001) | .69 (.017) | Met normality and homogeneity of variances for all conditions |
| Spring All | .13 (.001) | .65 (.006) | .13 (.001) | Failed normality (except other race) and homogeneity of variance |
| Spring Matched | .36 (.005) | .51 (.014) | .35 (.009) | Failed normality (except other race) |
| Combined All | .11 (.000) | .27 (.001) | .21 (.001) | Failed normality and homogeneity of variance for all conditions |
| Combined Matched | .15 (.001) | .32 (.004) | .07 (.001) | Failed normality (except other race) |

**p* < .05, ***p* < .01

**Subscales Favorability Change**

| Cohort | F1: Real World Connection  (*t*) | F2: Problem Solving Difficulty  (*t*) | F3: Enjoyment  (*t*) | F4: Problem Solving Effort  (*t*) | F5: Conceptual Connections & Memorization  (*t*) | F6: Problem Solving Strategies  (*t*) | F7: Reasoning (*t*) |
| --- | --- | --- | --- | --- | --- | --- | --- |
| Fall All^1^ | .18 | 1.42 | .46 | .88 | .48 | .70 | .62 |
| Fall Matched^2^ | .41 | .29 | .52 | .25 | 1.38 | .46 | .09 |
| Spring All^3^ | 2.25* | 2.01* | 1.51 | 1.03 | 2.26* | .44 | 1.44 |
| Spring Matched^4^ | 3.22** | 1.64 | 1.94 | 1.38 | 2.57* | .37 | 1.81 |
| Combined All^5^ | 1.73 | 2.44* | .80 | .15 | 1.41 | .17 | .65 |
| Combined Matched^6^ | 2.51* | 1.25 | .99 | .76 | .92 | .07 | 1.13 |
| **p* < .05, ***p* < .01  ^1^Failed normality (except intervention for F4)  ^2^Failed normality (except F4, F5 control)  ^3^Failed normality (except intervention on F1, F4, and F5)  ^4^Failed normality (except intervention on F1, F2, F5; control on F2)  ^5,6^Failed normality | | | | | | | |

**Subscales Favorability Change: Covariates**

| Cohort | Subscales: F1-F7  Sex, Race, Sex *Race |
| --- | --- |
| Fall All | F6 Sex (Male: 8.19, Female -2.01) – violated normality for m/f |
| Fall Matched | F6 Sex (Male: 9.29, Female -4.90) – violated normality for m/f |
| Spring All | No significant differences |
| Spring Matched | No Significant differences |
| Combined All | No significant differences |
| Combined Matched | No significant differences |

-Bonferonni used for race in post-hoc analyses

**Lab skills**

**Fall 2017 Matched Cohort**

| **Multivariate Tests^a^** | | | | | | | |
| --- | --- | --- | --- | --- | --- | --- | --- |
| Effect | | Value | F | Hypothesis df | Error df | Sig. | Partial Eta Squared |
| Intercept | Pillai's Trace | .304 | 2.220^b^ | 14.000 | 71.000 | .015 | .304 |
|  | Wilks' Lambda | .696 | 2.220^b^ | 14.000 | 71.000 | .015 | .304 |
|  | Hotelling's Trace | .438 | 2.220^b^ | 14.000 | 71.000 | .015 | .304 |
|  | Roy's Largest Root | .438 | 2.220^b^ | 14.000 | 71.000 | .015 | .304 |
| GROUP | Pillai's Trace | .361 | 2.861^b^ | 14.000 | 71.000 | .002 | .361 |
|  | Wilks' Lambda | .639 | 2.861^b^ | 14.000 | 71.000 | .002 | .361 |
|  | Hotelling's Trace | .564 | 2.861^b^ | 14.000 | 71.000 | .002 | .361 |
|  | Roy's Largest Root | .564 | 2.861^b^ | 14.000 | 71.000 | .002 | .361 |
| a. Design: Intercept + SEMESTER + GROUP + SEMESTER * GROUP | | | | | | | |
| b. Exact statistic | | | | | | | |

There was a significant main effect of group on student’s change in confidence *F*(14,71) = 2.222, *p* = .002, $\eta^{2}$ = .361. Students in the intervention group reported significantly greater levels of improvement in confidence than did students in the control group.

Significant differences (highlighted) between control and intervention groups were evident for the following questions:

| Source | Dependent Variable | Type III Sum of Squares | df | Mean Square | F | Sig. | Partial Eta Squared |
| --- | --- | --- | --- | --- | --- | --- | --- |
| GROUP | Q1_CHG | .000 | 1 | .000 | .000 | 1.000 | .000 |
|  | Q2_CHG | 1.407 | 1 | 1.407 | 1.203 | .276 | .014 |
|  | Q3_CHG | .000 | 1 | .000 | .000 | 1.000 | .000 |
|  | Q4_CHG | 7.267 | 1 | 7.267 | 7.902 | .006 | .086 |
|  | Q5_CHG | 11.907 | 1 | 11.907 | 9.744 | .002 | .104 |
|  | Q6_CHG | 4.651 | 1 | 4.651 | 4.238 | .043 | .048 |
|  | Q7_CHG | 11.174 | 1 | 11.174 | 6.314 | .014 | .070 |
|  | Q8_CHG | 18.605 | 1 | 18.605 | 12.542 | .001 | .130 |
|  | Q9_CHG | 2.279 | 1 | 2.279 | 1.797 | .184 | .021 |
|  | Q10_CHG | .186 | 1 | .186 | .153 | .697 | .002 |
|  | Q11_CHG | .291 | 1 | .291 | .239 | .626 | .003 |
|  | Q12_CHG | 3.767 | 1 | 3.767 | 2.342 | .130 | .027 |
|  | Q13_CHG | .105 | 1 | .105 | .095 | .758 | .001 |
|  | Q14_CHG | .291 | 1 | .291 | .135 | .715 | .002 |

**Spring 2018 Matched Cohort**

**Multivariate Tests^a^**

|  | | | | | | | |
| --- | --- | --- | --- | --- | --- | --- | --- |
| Effect | | Value | F | Hypothesis df | Error df | Sig. | Partial Eta Squared |
| Intercept | Pillai's Trace | .442 | 3.671^b^ | 14.000 | 65.000 | .000 | .442 |
|  | Wilks' Lambda | .558 | 3.671^b^ | 14.000 | 65.000 | .000 | .442 |
|  | Hotelling's Trace | .791 | 3.671^b^ | 14.000 | 65.000 | .000 | .442 |
|  | Roy's Largest Root | .791 | 3.671^b^ | 14.000 | 65.000 | .000 | .442 |
| GROUP | Pillai's Trace | .296 | 1.952^b^ | 14.000 | 65.000 | .036 | .296 |
|  | Wilks' Lambda | .704 | 1.952^b^ | 14.000 | 65.000 | .036 | .296 |
|  | Hotelling's Trace | .420 | 1.952^b^ | 14.000 | 65.000 | .036 | .296 |
|  | Roy's Largest Root | .420 | 1.952^b^ | 14.000 | 65.000 | .036 | .296 |
| a. Design: Intercept + SEMESTER + GROUP + SEMESTER * GROUP | | | | | | | |
| b. Exact statistic | | | | | | | |

There was a significant main effect of group on student’s change in confidence *F*(14,65) = 1.952, *p* = .036, $\eta^{2}$ = .296. Students in the intervention group reported significantly greater levels of improvement in confidence than did students in the control group.

Significant differences (highlighted) between control and intervention groups were evident for the following questions:

| Source | Dependent Variable | Type III Sum of Squares | df | Mean Square | F | Sig. | Partial Eta Squared |
| --- | --- | --- | --- | --- | --- | --- | --- |
| GROUP | Q1_CHG | 3.200 | 1 | 3.200 | 4.732 | .033 | .057 |
|  | Q2_CHG | 2.813 | 1 | 2.813 | 2.235 | .139 | .028 |
|  | Q3_CHG | 1.250 | 1 | 1.250 | .995 | .322 | .013 |
|  | Q4_CHG | 8.450 | 1 | 8.450 | 7.709 | .007 | .090 |
|  | Q5_CHG | 18.050 | 1 | 18.050 | 15.320 | .000 | .164 |
|  | Q6_CHG | .113 | 1 | .113 | .108 | .743 | .001 |
|  | Q7_CHG | .613 | 1 | .613 | .574 | .451 | .007 |
|  | Q8_CHG | 13.613 | 1 | 13.613 | 10.526 | .002 | .119 |
|  | Q9_CHG | .450 | 1 | .450 | .526 | .471 | .007 |
|  | Q10_CHG | .800 | 1 | .800 | .891 | .348 | .011 |
|  | Q11_CHG | 2.113 | 1 | 2.113 | 2.428 | .123 | .030 |
|  | Q12_CHG | 7.200 | 1 | 7.200 | 5.528 | .021 | .066 |
|  | Q13_CHG | 2.450 | 1 | 2.450 | 2.663 | .107 | .033 |
|  | Q14_CHG | 4.513 | 1 | 4.513 | 3.118 | .081 | .038 |

**Combined Fall and Spring Matched Cohorts**

| **Multivariate Tests^a^** | | | | | | | |
| --- | --- | --- | --- | --- | --- | --- | --- |
| Effect | | Value | F | Hypothesis df | Error df | Sig. | Partial Eta Squared |
| Intercept | Pillai's Trace | .328 | 5.189^b^ | 14.000 | 149.000 | .000 | .328 |
|  | Wilks' Lambda | .672 | 5.189^b^ | 14.000 | 149.000 | .000 | .328 |
|  | Hotelling's Trace | .488 | 5.189^b^ | 14.000 | 149.000 | .000 | .328 |
|  | Roy's Largest Root | .488 | 5.189^b^ | 14.000 | 149.000 | .000 | .328 |
| SEMESTER | Pillai's Trace | .072 | .826^b^ | 14.000 | 149.000 | .640 | .072 |
|  | Wilks' Lambda | .928 | .826^b^ | 14.000 | 149.000 | .640 | .072 |
|  | Hotelling's Trace | .078 | .826^b^ | 14.000 | 149.000 | .640 | .072 |
|  | Roy's Largest Root | .078 | .826^b^ | 14.000 | 149.000 | .640 | .072 |
| GROUP | Pillai's Trace | .274 | 4.023^b^ | 14.000 | 149.000 | .000 | .274 |
|  | Wilks' Lambda | .726 | 4.023^b^ | 14.000 | 149.000 | .000 | .274 |
|  | Hotelling's Trace | .378 | 4.023^b^ | 14.000 | 149.000 | .000 | .274 |
|  | Roy's Largest Root | .378 | 4.023^b^ | 14.000 | 149.000 | .000 | .274 |
| SEMESTER * GROUP | Pillai's Trace | .108 | 1.293^b^ | 14.000 | 149.000 | .218 | .108 |
|  | Wilks' Lambda | .892 | 1.293^b^ | 14.000 | 149.000 | .218 | .108 |
|  | Hotelling's Trace | .121 | 1.293^b^ | 14.000 | 149.000 | .218 | .108 |
|  | Roy's Largest Root | .121 | 1.293^b^ | 14.000 | 149.000 | .218 | .108 |
| a. Design: Intercept + SEMESTER + GROUP + SEMESTER * GROUP | | | | | | | |
| b. Exact statistic | | | | | | | |

There was no significant interaction between semester and group on student’s change in confidence *F*(14,149) = 1.29, *p* = .218, $\eta^{2}$ = .108.

There was no significant main effect of semester on student’s change in confidence *F*(14,149) = .826, *p* = .64, $\eta^{2}$ = .072.

There was a significant main effect of group on student’s change in confidence *F*(14,149) = 4.02, *p* < .001, $\eta^{2}$ = .274. Students in the intervention group reported significantly greater levels of improvement in confidence than did students in the control group.

Significant differences (highlighted) between control and intervention groups were evident for the following questions:

| Source | Dependent Variable | Type III Sum of Squares | df | Mean Square | F | Sig. | Partial Eta Squared |
| --- | --- | --- | --- | --- | --- | --- | --- |
| GROUP | Q1_CHG | 1.658 | 1 | 1.658 | 2.296 | .132 | .014 |
|  | Q2_CHG | 4.123 | 1 | 4.123 | 3.401 | .067 | .021 |
|  | Q3_CHG | .648 | 1 | .648 | .510 | .476 | .003 |
|  | Q4_CHG | 15.711 | 1 | 15.711 | 15.638 | .000 | .088 |
|  | Q5_CHG | 29.740 | 1 | 29.740 | 24.764 | .000 | .133 |
|  | Q6_CHG | 1.577 | 1 | 1.577 | 1.473 | .227 | .009 |
|  | Q7_CHG | 8.317 | 1 | 8.317 | 5.809 | .017 | .035 |
|  | Q8_CHG | 31.922 | 1 | 31.922 | 22.935 | .000 | .124 |
|  | Q9_CHG | 2.344 | 1 | 2.344 | 2.191 | .141 | .013 |
|  | Q10_CHG | .890 | 1 | .890 | .838 | .361 | .005 |
|  | Q11_CHG | 2.018 | 1 | 2.018 | 1.923 | .167 | .012 |
|  | Q12_CHG | 10.751 | 1 | 10.751 | 7.357 | .007 | .043 |
|  | Q13_CHG | .814 | 1 | .814 | .804 | .371 | .005 |
|  | Q14_CHG | 3.622 | 1 | 3.622 | 1.995 | .160 | .012 |

**Correlations between Lab report and Lab exam**

**Correlation between Lab exam forced choice and Lab report average sore**

|  | | Lab report  Average score between raters |
| --- | --- | --- |
| Lab exam Forced Choice Sum | Pearson Correlation | .531^**^ |
|  | Sig. (2-tailed) | .000 |
|  | N | 81 |
| **. Correlation is significant at the 0.01 level (2-tailed). | | |

There was a significant correlation between Lab exam written work and average Lab report score (*r* = .531, *p* < .001).

**Correlation between Lab exam written work and Lab report average score**

|  | | Lab report  Average score between raters |
| --- | --- | --- |
| Lab exam Written: Rater Average | Pearson Correlation | .495^**^ |
|  | Sig. (2-tailed) | .000 |
|  | N | 81 |
| **. Correlation is significant at the 0.01 level (2-tailed). | | |

There was a significant correlation between Lab exam written work and average Lab report score (*r* = .495, *p* < .001).

**Correlation between Lab exam written work and forced choice.**

|  | | Lab exam Forced Choice Sum |
| --- | --- | --- |
| Lab exam Written: Rater Average | Pearson Correlation | .596^**^ |
|  | Sig. (2-tailed) | .000 |
|  | N | 81 |
| **. Correlation is significant at the 0.01 level (2-tailed). | | |

There was a significant correlation between Lab exam written work and forced choice responses (*r* = .596, *p* < .001).

**Interrater reliability**

**Lab report**

| **Descriptive Statistics** | | | |
| --- | --- | --- | --- |
|  | Mean | Std. Deviation | N |
| SUM1 | 13.9756 | 1.93075 | 82 |
| SUM2 | 14.1220 | 1.95246 | 82 |

| **Correlations** | | |
| --- | --- | --- |
|  | | SUM2 |
| SUM1 | Pearson Correlation | .764^**^ |
|  | Sig. (2-tailed) | .000 |
|  | N | 82 |
| **. Correlation is significant at the 0.01 level (2-tailed). | | |

**Lab exam**

| **Correlations** | | |
| --- | --- | --- |
|  | | FA Written: Rater 2 Sum |
| FA Written: Rater 1 Sum | Pearson Correlation | .798^**^ |
|  | Sig. (2-tailed) | .000 |
|  | N | 81 |
| **. Correlation is significant at the 0.01 level (2-tailed). | | |

There was a significant correlation between rater 1 and rater 2 on the Lab exam written choice (*r* = .798, *p* < .001).

**E-EDAT**

Interrater reliability between Rater 1 and Rater 2 (all judges combined)

| **Cohort** | **Pearson Correlation (*r*)** | **N** |
| --- | --- | --- |
| Fall | .734*** | 84 |
| Spring | .77*** | 79 |
| Combined | .749*** | 163 |
| ***Correlation significant at the *p* < .001 level (2-tailed) | | |

**Reflections**

|  |  |  |  |
| --- | --- | --- | --- |
| **Subscale** | **Rater 1 mean (*sd*)** | **Rater 2 mean (*sd*)** | **Correlation (*r*)** |
| Interesting | 2.01 (1.05) | 2.19 (1.05) | .79*** |
| Valuable | 1.43 (.90) | 1.57(1.02) | .82*** |
| Difficulty | 1.13 (.58) | 1.25 (.57) | .62*** |
| Science Process | 1.34 (.94) | 1.02 (.76) | .60*** |
| Overall Labs | 1.61 (.49) | 1.51 (.51) | .55*** |
| Sum (all scales) | 7.52 (2.41) | 7.54 (2.21) | .82*** |

Interrater correlations on all subscales and sum score were significant (*p* < .001), indicating a high degree of interrater reliability.
